# Seasonal Coloration and Ecological Adaptations of Adventitious Roots of Four Salicaceous Species in Jiuzhaigou World Natural Heritage Site, Southwestern China

**DOI:** 10.1002/ece3.71218

**Published:** 2025-04-15

**Authors:** Ting Liu, Junhuai Xu, Weiyang Xiao, Lv Zhou, Yingzhou Chen, Xue Qiao, Sha Deng, Zongliang Du, Ya Tang

**Affiliations:** ^1^ Institute of New Energy and Low‐Carbon Technology Sichuan University Chengdu Sichuan China; ^2^ College of Architecture and Environment Sichuan University Chengdu Sichuan China; ^3^ China Southwest Architecture Design and Research Institute Co. Ltd. CSCEC Green Construction Engineering Research Center Chengdu Sichuan China; ^4^ College of Biomass Science and Engineering Sichuan University Chengdu Sichuan China; ^5^ Jiuzhaigou Administrative Bureau Zhangzha Jiuzhaigou, Sichuan China

**Keywords:** Jiuzhaigou, proanthocyanidins, red adventitious roots, seasonal coloration, tufa wetland

## Abstract

Jiuzhaigou is a world natural heritage with extraordinary beauty of wetlands largely developed on tufa landforms. The wetlands are dominated by shrubs and trees. A striking feature of dense and plentiful adventitious roots is found during summer, and the color changes to unnoticeable during winter. Despite the visual prominence of this phenomenon, its biochemical mechanisms and ecological significance remain unexplored. Integrating field surveys, anatomical analyses, and biochemical profiling to decipher coloration dynamics and their potential as environmental bioindicators, results indicate that dense adventitious roots were found only with willow and poplar species in the tufa wetlands in Shuzheng and Rize valleys. Adventitious roots displayed specialized adaptations, including well‐developed aerenchyma, degenerated mechanical tissue and xylem, and a floating habit on the water surface, which enhances oxygen uptake in aquatic habitats. Seasonal color variations followed a distinct temporal pattern, transitioning from red or pink hues in summer to reddish‐brown in spring and autumn, and maroon or gray in winter. Proanthocyanidins were identified as principal pigments, with their oxidation into quinones under the influence of temperature and light driving the observed color transitions. The proanthocyanidins redox dynamics reflect seasonal fluctuations in air temperature and solar irradiance, providing a novel biomarker for assessing climate impacts on wetland ecosystems. The close link between seasonal color change of adventitious roots and the aquatic environment sheds new light on effective ecosystem management in karst areas.

## Graphical abstract

This study examines the seasonal coloration and ecological adaptations of adventitious roots in four Salicaceae species at Jiuzhaigou World Natural Heritage Site, revealing their morphological, anatomical, and pigment composition characteristics. The research identifies the red coloration in warm seasons as proanthocyanidins, potentially serving as a bioindicator for environmental changes.

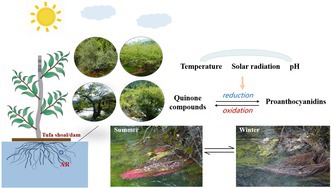

## 1 Introduction

Wetlands are among the most biodiverse ecosystems on earth, providing essential services such as water purification, flood control, and climate change mitigation (Mao et al. 2023; Jiang et al. 2024; Yao et al. 2022). Jiuzhaigou valley (hereafter referred to as Jiuzhaigou) has been designated as a World Natural Heritage Site by UNESCO for its exceptional karst tufa landscapes (Li et al. 2020; Deng et al. 2020). This region is one of the most biodiverse wetland areas on the Tibetan Plateau and supports unique wetland systems dominated by woody plant species adapted to the tufa landform (Jiao et al. 2023). However, these wetlands are threatened by degradation due to natural and anthropogenic activities, including climate warming, atmospheric deposition, hydrochemical changes, and others (Qiao et al. 2022; Zheng et al. 2022). This degradation not only affects the esthetic value of the region but also threatens the ecological stability and biodiversity of the wetland. Therefore, understanding the adaptive mechanisms of the dominant plant species is essential for developing effective conservation strategies.

**FIGURE 1:**
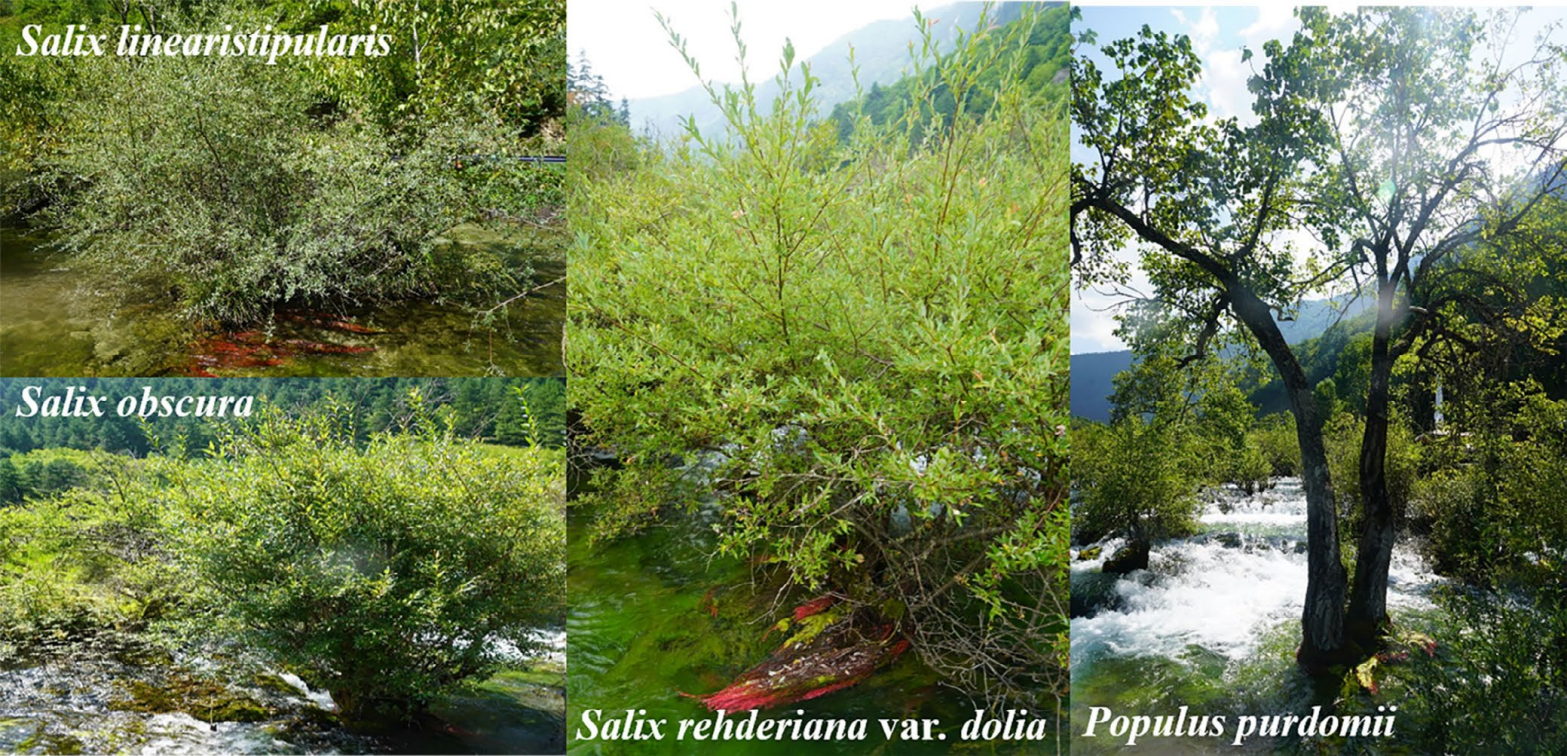
Photographs of the four study species.

In these wetland systems, there is a striking feature of abundant adventitious roots (ARs) that float in water, turning a vibrant red hue during the warm season and undergoing seasonal color changes. Field investigations have revealed that ARs are found only on three willow and one poplar species, namely, *Salix obscura*, *S. linearistipularis*, *S. rehderiana* var. *dolia*, and *Populus purdomii* (Figure 1). They are also the dominant species in the tufa wetlands of Jiuzhaigou. However, the extensive and abundant red ARs have received little research attention, and the mechanisms of this striking red coloration and their potential utility as a biological indicator remain unknown. At present, studies on wetland plants have extensively explored their physiological and biochemical responses to environmental changes, plant‐microbe interactions, and ecological restoration (Sati et al. 2025; Wang et al. 2023; Zhang et al. 2023). Research on color responses has mainly focused on the pigmentation mechanisms of flowers and leaves, while adventitious roots in wetlands are relatively scarce (Gonin et al. 2019; Zhang et al. 2019; Zhao et al. 2022). The unique environmental conditions of the tufa wetlands in Jiuzhaigou offer a valuable opportunity to explore their color adaptation strategies. This research holds broader implications for the conservation of global karst landscapes.

The objectives of the study are to understand the biological adaptation mechanisms of ARs in Jiuzhaigou's tufa wetlands and to reveal the underlying reasons for the striking red coloration. The hypotheses of this study are: (1) the growth of ARs is an adaptation to the waterlogged environment of the tufa wetlands; (2) the coloration of ARs is linked to pigments and color changes are associated with alterations in the internal compound structure of the ARs; and (3) the transformation of natural products in the roots is related to temperature and light intensity. The results may provide essential data for developing sustainable ecosystem management and conservation strategies. Furthermore, this study can help prevent the degradation of tufa wetlands and preserve the health of the tufa wetland landscape.

## 2 Materials and Methods

### 2.1 Study Area

**FIGURE 2:**
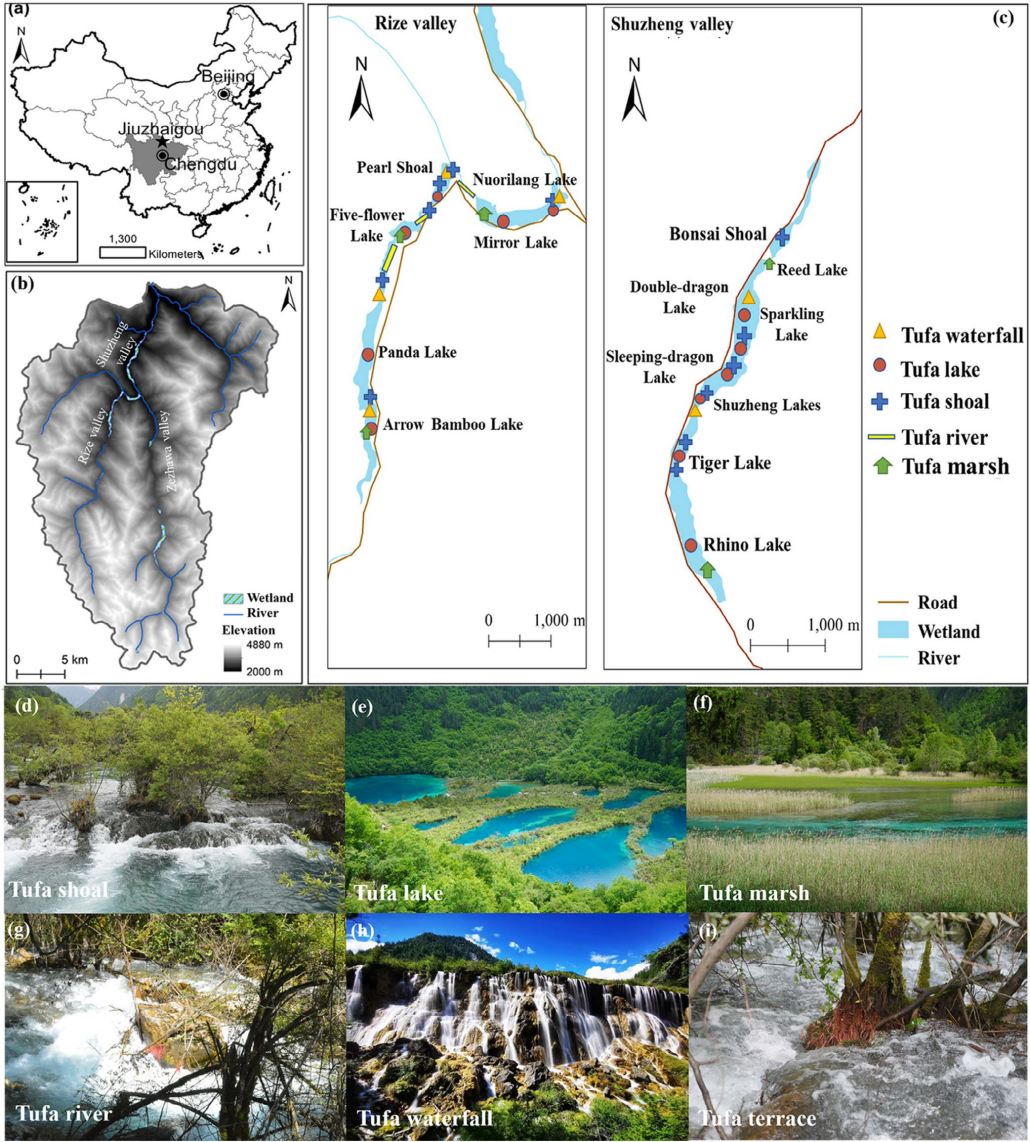
Location of Jiuzhaigou in southwestern China (a), its wetland types, distribution (b, c), and tufa landscape (d–i).

Lying in the transitional areas from the lowland Sichuan Basin to the highland Tibetan Plateau, Jiuzhaigou is widely known for its exceptional beauty of lakes, pools, and waterfalls at the bottom and dense forests on both sides of the valleys in a watershed hidden in the mountains. It covers an area of 643 km^2^, with an elevation ranging from 1996 to 4764 m above sea level (a.s.l.) (Figure 2). The mean annual temperature is 7.1°C, with the highest in July at 23.3°C and the lowest in January at −10.3°C. The mean annual precipitation is 703 mm, with obvious seasonal and annual variations and mostly falling from April to October. The seasonal variations in precipitation cause considerable changes in surface water quantity. Therefore, the surface water level within the catchment is highest in October and lowest in April (Laffitte 2021). The surface water is alkaline, with a pH value of 7.30–8.50. The main ions are Ca^2+^ (36.1–87.2 mg/L) and Mg^2+^ (4.91–20.7 mg/L), and the main anions are SO_4_^2−^ (3.50–36.1 mg/L) and HCO_3_^−^ (134–281 mg/L) (Qiao et al. 2022).

The main picturesque tourist attractions include various wetlands. There are 21 types of wetlands, covering an area of 265 ha, with 42.8% on tufa (Du et al. 2018). Tufa is a type of porous calcite precipitated from Ca^2+^‐rich water when CO_2_ degases from water in the ambient environment. Tufa landscapes mainly spread in the bottoms of the Rize and Shuzheng valleys (Du et al. 2018).

Wetland plant communities are distributed in the valley bottom mainly from 2000 to 2900 m a.s.l., largely dominated by *Salix* species. They form distinct belt vegetation along the tufa shoals and dams in the Shuzheng and Rize valleys (Figure 2e). Other woody species include *Sorbaria arborea* , *Philadelphus incanus*, *Berberis diaphana*, *Lonicera acuminata* , *Rosa bella* , *Sorbus vilmorinii*, *Fargesia nitida* , *Deutzia longifolia*, *Hydrangea macrophylla* , *Potentilla fruticosa* , *Taxus wallichiana* var. *chinensis*, *Acer pictum* subsp. *mono*, and *Viburnum betulifolium*. Common herbaceous species are *Hippuris vulgaris* , *Equisetum arvense* , *Potamogeton perfoliatus* , *Equisetum hyemale* , *Phragmites australis* , and *Typha latifolia* .

### 2.2 Spatial Distribution of Plants With ARs and Seasonal Color Change of ARs

To investigate the spatial occurrence of ARs, a systematic sampling approach was applied. Transect lines were positioned at 50 m intervals throughout the wetland areas. Along each transect, a plot of 20 m × 20 m was selected at 20 m intervals. Each plot was further divided into four equal quadrats of 5 m × 5 m (Figure S1). Within each quadrat, we recorded plant species, the proportion of plants within ARs, and the length of ARs.

As ARs undergo color changes after harvesting, color changes in ARs were assessed in situ through repeat photography conducted once a month for the above‐mentioned four species from March 2021 to February 2022. Their spatial distribution and locations are detailed in Table S1. The photographic setup comprised a Sony Alpha 7R III camera paired with a Vario‐Tessar T* FE 24‐70 mm F4 ZA OSS lens and a Nikon D7100 camera equipped with an AF‐S 24‐85 mm f/3.5–4.5G ED VR lens. For quantitative color analysis, we utilized Adobe Photoshop in CIE Lab color mode (Kulapichitr et al. 2022). This method allows for precise measurement of luminosity (L*), red/green (a*), and yellow/blue (b*) parameters at the meristem region, elongation region, and maturation region of ARs. Mean values were used to characterize the color at each region. Chromaticity (C*) was conversed in CIE Lab using the following formula:

$$C^{*}={\left(a^{*2}+b^{*2}\right)}^{1/2}$$

At each site (Table S2), water temperature, pH, dissolved oxygen (DO), conductivity, and concentrations of Ca^2+^, Mg^2+^, K^+^, Na^+^, F^−^, Cl^−^, NO_3_^−^, SO_4_^2−^, and TN were measured once a month by using the methods and instruments listed in Table S3. The HCO_3_^−^ concentration was calculated using the PHREEQC model (Parkhurst and Appelo 2013). Moreover, the meteorological data (air temperature, solar radiation, and precipitation) were obtained from the Zezhawa Meteorological Station (33°16′ N, 103°91′ E, 2414 m a.s.l.).

### 2.3 Anatomical Characteristics of ARs

Due to the difficulty in sampling from *Populus purdomii*, AR samples were collected only from three healthy *Salix* species in the primary tufa wetland. Detailed sample information is provided in Table S4. Then, approximately 1–2 cm of root tips were excised and fixed in FAA (70% (v/v) ethanol: acetic acid: formalin = 90:5:5). The conventional paraffin section method was used to make sections. Serial sections were cut 10–12 μm thick, stained with saffron‐fast green, and sealed with neutral balsam. Finally, the sections were observed under a microscope (Axioskop, ZEISS) and photographed by an attached digital camera.

### 2.4 Extraction and Identification of Pigments

#### 2.4.1 Plant Materials and Chemicals

AR samples from three healthy *Salix* species were collected at the same location where samples for anatomical study were collected (Table S4). They were collected in December 2021 and August 2022, and stored in a container filled with water from Jiuzhaigou at −20°C before further lab analysis.

Standard substances, including catechin (C, ≥ 98%), epicatechin (EC, ≥ 98%), gallocatechin (GC, ≥ 98%), epigallocatechin (EGCG, ≥ 98%), and L‐Ascorbic acid (≥ 98%), and HPLC‐grade methanol and acetonitrile were purchased from Beijing Solarbio Science & Technology Co. Ltd. (Beijing China). Analytical grade ethanol and potassium bromide were purchased from Sinopharm Chemical Reagent Co. Ltd. (Shanghai, China).

#### 2.4.2 Extraction of Pigments

Extraction method followed Tava et al. (2021) and Pals et al. (2020), with slight modifications. Samples were freeze‐dried (SCIENTZ‐18N, Ningbo, China) and pulverized (Molino Pulvex Mini 100) through a 60‐mesh sieve. The finely powdered and dried materials were light‐sealed at room temperature (25°C) before use. About 3 g of dry powder was added to 45 mL of 60% ethanol (with a solid–liquid ratio of 1:15), heated in a water bath at 85°C for two hours, and repeated twice. The mixture was vacuum filtered, and the extract was combined and concentrated by a rotary evaporator (N‐1100‐D, GYYQ) to obtain a crude extract. Subsequently, 50 mL of 6% sulfurous acid was added to the crude extract, and the mixture was refluxed at 85°C for two hours. After cooling, the mixture was centrifuged to remove the supernatant, and deionized water was added several times to dilute the remaining acid until the pH reached neutrality. The resulting solid was freeze‐dried and stored at 4°C until use.

#### 2.4.3 Identification of Pigments

1. UV–visible spectra

UV–visible spectra were obtained with an Ultraviolet–visible Spectrometer (Specord S600, Analytik Jena). All measurements were conducted at room temperature using a 1 cm path length cuvette. Root extracts were dissolved in MeOH, and spectra were recorded against the corresponding solvent as the baseline in the spectral range between 200 and 800 nm with a spectral resolution of 1 nm.

2 Infrared spectra

The extracted powder samples were mixed with KBr, ground, dried, and pressed into pellets for Fourier Transform Infrared Spectrometer (FTIR, Nicolet NEXUS‐670) analysis. FTIR spectra of each sample were scanned within the wavelength range of 4000 to 400 cm^−1^.

3 LC–MS analysis

As color changes were the same among the three species, we used samples of one species (*Salix obscura*) to analyze its color changes, following Pals et al. (2020) with modifications according to the specific conditions of this research. All samples were filtered through a 0.22 μm organic membrane filter and analyzed using liquid chromatography‐mass spectrometry (LC–MS) (Thermo Fisher, USA). An injection volume of 2 μL was used in the C_18_ analytical column (Capcell Pak C_18_ MGII S5, 250 × 4.6 mm) at an oven temperature of 30°C. Solvent A (CH_3_CN: CH_3_COOH: H_2_O = 90:20:890) and solvent B (CH_3_CN: CH_3_COOH: H_2_O = 800:20:180) were utilized as eluents with the following gradient: 0–10 min, 0% B; 10–30 min, 25% B; 30–36 min, 0% B. The column was washed and reconditioned. Elution was monitored at 280 nm using a dual‐channel UV–visible light detector, and the flow rate was maintained at 1.0 mL/min. All mass spectrometry (MS) experiments were conducted in negative ion mode [M−H]^−^, using nitrogen as the nebulizing gas and helium as the damping gas. The ion source parameters were set with a spray voltage of 5.0 kV, a nebulizer gas pressure of 35 psi, a capillary voltage of 90.0 V, and a capillary temperature of 350°C. Data acquisition and processing were performed using MS workstation software (Version 6.9). Full scan mode was employed for data collection over the *m*/*z* range of 50 to 2000. MS/MS analyses were conducted on selected precursor ions (Aguilar et al. 2017) with a collision energy level of 35%.

4 Effects of pH, temperature, and light intensity on photoirradiation

Equal quantity of extracts was added into test tubes, and the pH value was adjusted using 1 mol/L HCl and 1 mol/L NaOH to observe changes in the color of the extracts.

We exposed AR extracts to a photoirradiation process to simulate naturally occurring phenomena to induce photooxidative reactions. The extracts were irradiated with LED lights (wavelength ranges of 450–495 nm and 620–750 nm) and UV‐A ultraviolet (wavelength range of 320–400 nm) to observe color change over time, with four treatments: dark and low temperature (T1), low‐temperature photoirradiation (T2), room‐temperature photoirradiation (T3), and high‐intensity photoirradiation at room temperature (T4). T1 extracts were preserved in dark glass bottles at 4°C. For other treatments, based on the daily solar radiation intensity variations of Jiuzhaigou (Figure S2), the LED light exposure protocols followed Table 1. The LED light source was fixed 15 cm above the samples to ensure uniform exposure throughout the 5‐day experimental period. Subsequently, the irradiated products were analyzed using UV spectroscopy and LC–MS.

**TABLE 1:** Photoperiod and light intensity of four treatments.

	Time	Temperature (°C)	pH	UV‐A (320–400 nm) (W/m^2^)	Blue‐LED (450–495 nm) (W/m^2^)	Red‐LED (620–750 nm) (W/m^2^)
T1	All day	4	8.3	0	0	0
	07:00‐09:00	4	8.3	80	160	80
	09:00‐10:00	4	8.3	160	320	160
	10:00‐15:00	4	8.3	320	560	320
T2	15:00‐16:00	4	8.3	240	480	240
	16:00‐19:00	4	8.3	160	240	160
	19:00‐07:00	4	8.3	0	0	0
	07:00‐09:00	4	8.3	80	160	80
	09:00‐10:00	4	8.3	160	320	160
T3	10:00‐15:00	Room temperature (17°C–21°C)	8.3	320	560	320
	15:00‐16:00	Room temperature	8.3	240	480	240
	16:00‐19:00	Room temperature	8.3	160	240	160
	19:00‐07:00	Room temperature	8.3	0	0	0
	07:00‐09:00	Room temperature	8.3	160	320	160
	09:00‐10:00	Room temperature	8.3	320	480	320
T4	10:00‐15:00	Room temperature (17°C–21°C)	8.3	400	560	400
	15:00‐16:00	Room temperature	8.3	320	480	320
	16:00‐19:00	Room temperature	8.3	240	400	240
	19:00‐07:00	Room temperature	8.3	0	0	0

### 2.5 Data Processing and Statistical Analysis

All experiments were conducted with a minimum of three replicates. The arithmetic means and standard errors of replicates were calculated. A parametric one‐way analysis of variance (ANOVA) followed by a post hoc Tukey‐HSD test was used to determine differences among the various plant species. All statistical procedures were performed using IBM SPSS Statistics v.26.0 (IBM, NY, USA). Statistical significance was determined at *p* < 0.05 and *p* < 0.01. Pearson's correlation analysis was used for correlation assessments. Graphs were generated using Origin 2024.

## 3 Results

### 3.1 Ecological Adaptation of ARs

#### 3.1.1 Temporal Color Change of ARs

**FIGURE 3:**
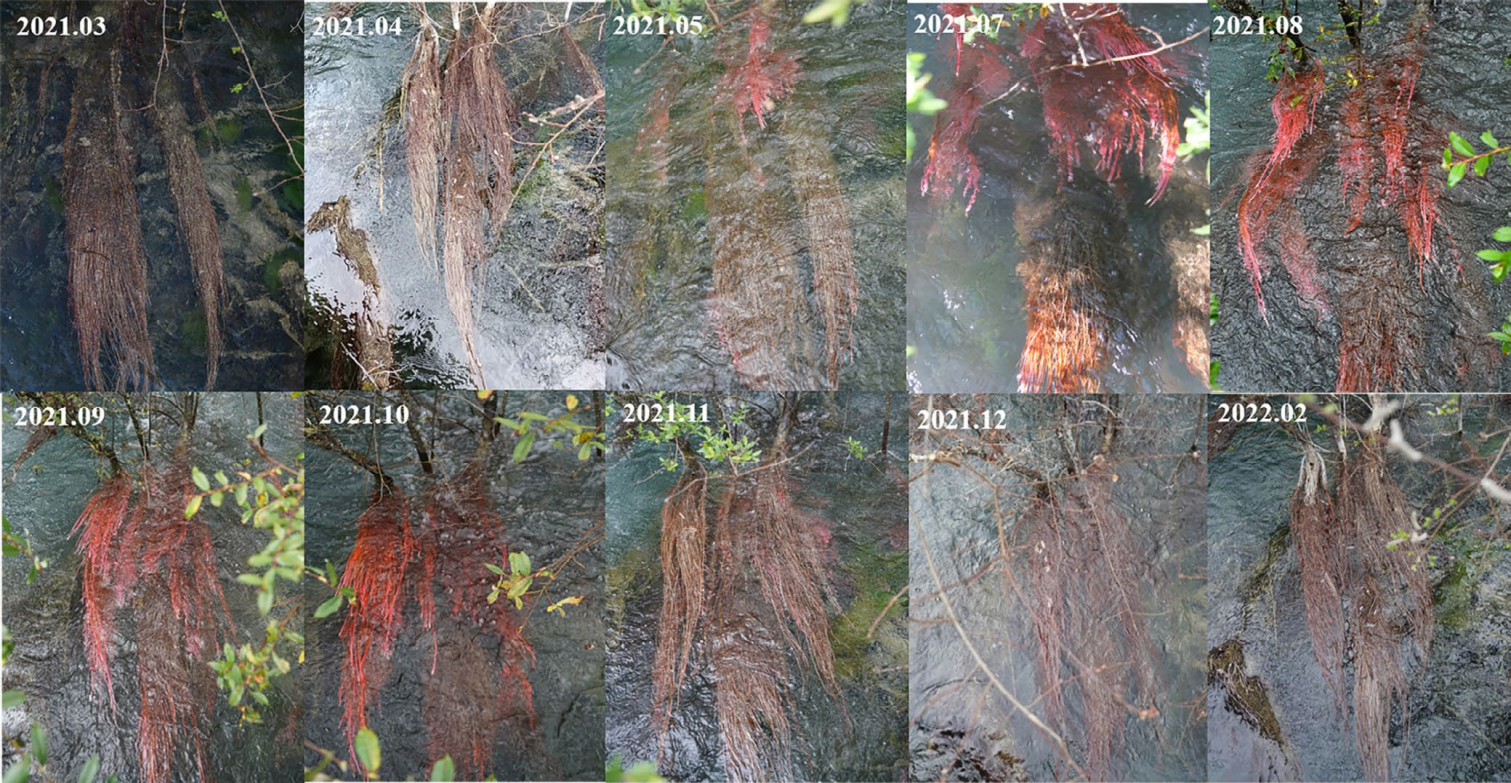
Monthly color changes in adventitious roots of *Salix obscura*.

Seasonal color changes in ARs of four species were evident (Figures 3 and S3–S5). As the colors in the photos could be affected by water level, lighting, and algal growth, we selected photos with the most explicit images and the most effortless color extraction for plant roots. The plant chosen for this analysis was *Salix obscura* from Shuzheng Lakes. From May to October, ARs were mostly red, pink, reddish‐brown, and orange, with those near the water surface being more vibrant. They turned red‐brown and brown from November to April, with those exposed above the water surface turning grayish‐white. Throughout the year, the ranges of L*, a*, b*, and C* values were 32–61, 7–64, 5–52, and 9.90–82.5, respectively (Table S5). All values of a* and b* were on the positive scales, suggesting that the ARs were red and yellow in the quadrant of Lab color space. Notably, the values of a*, b*, and C* increased in May and peaked in September (Figure S6), suggesting the formation of red and yellow‐pigmented substances.

**FIGURE 4:**
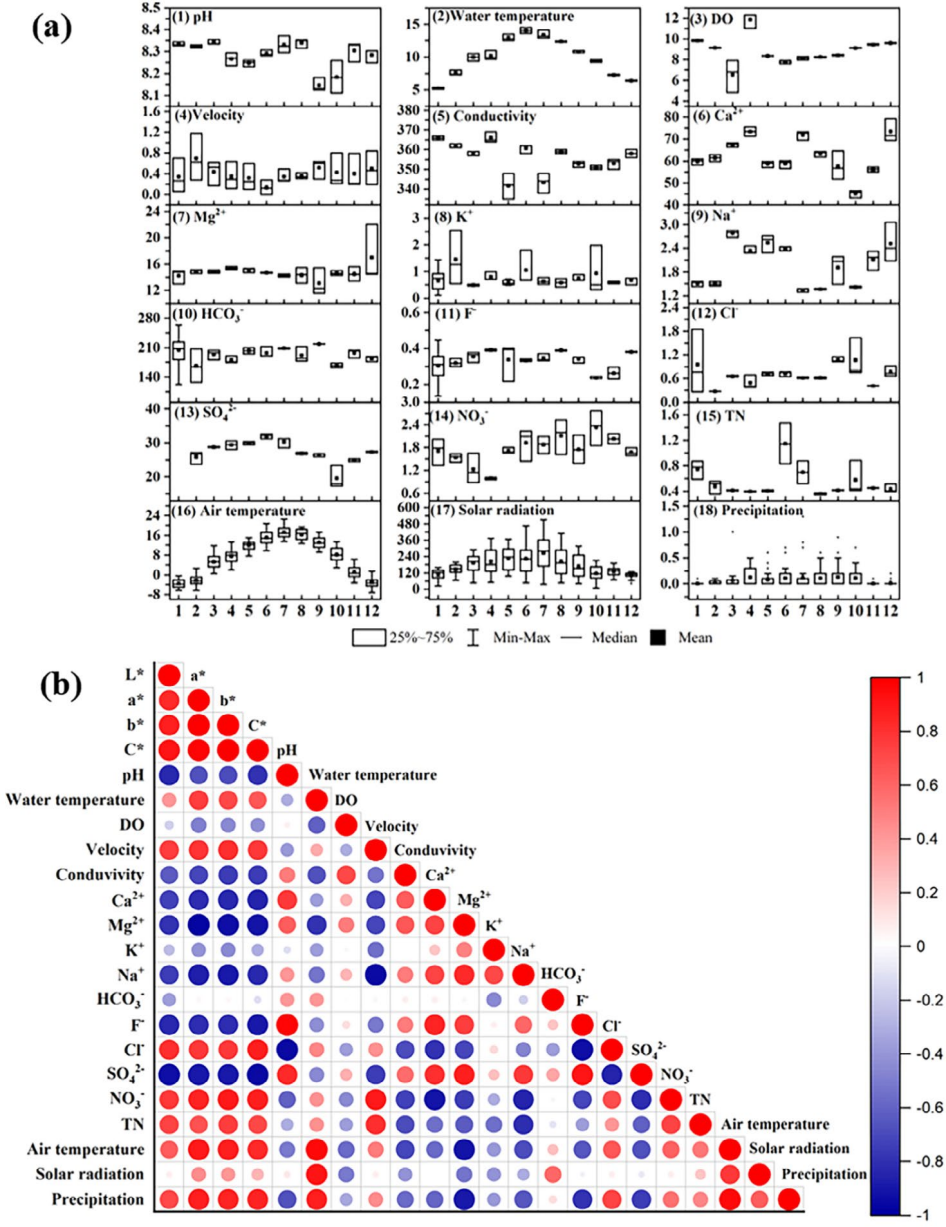
Monthly water chemistry variation and climatic factors in Shuzheng Lakes from March 2021 to February 2022 (a), and correlations between these factors and adventitious roots color (b). The unit for temperature, conductivity, velocity, alkalinity, solar radiation, and precipitation is °C, μS/cm, m/s, mg/L, W/m^2^, and mm, respectively. The unit for DO, ions, and TN is mg/L. Correlations range from −1 to 1. The closer to 1, the more positive the correlation, and the closer to −1, the more negative the correlation. For values close to 0, there is no correlation between the variables.

Analysis of water chemistry and meteorological data indicated that seasonal changes in solar radiation and temperature mirrored the color variations in ARs (Figure 4a). Correlation analysis revealed significant relationships between color changes and air temperature (*r*(a*) = 0.794, *p* < 0.01; *r*(b*) = 0.750, *p* < 0.05; *r*(C*) = 0.657, *p* < 0.05), precipitation (*r*(a*) = 0.672, *p* < 0.05; *r*(b*) = 0.659, *p* < 0.05; *r*(C*) = 0.683, *p* < 0.05), and NO_3_^−^ concentration (*r*(a*) = 0.664, *p* < 0.05; *r*(b*) = 0.742, *p* < 0.05; *r*(C*) = 0.651, *p* < 0.05) (Figure 4b).

#### 3.1.2 Spatial Distribution Characteristics

**FIGURE 5:**
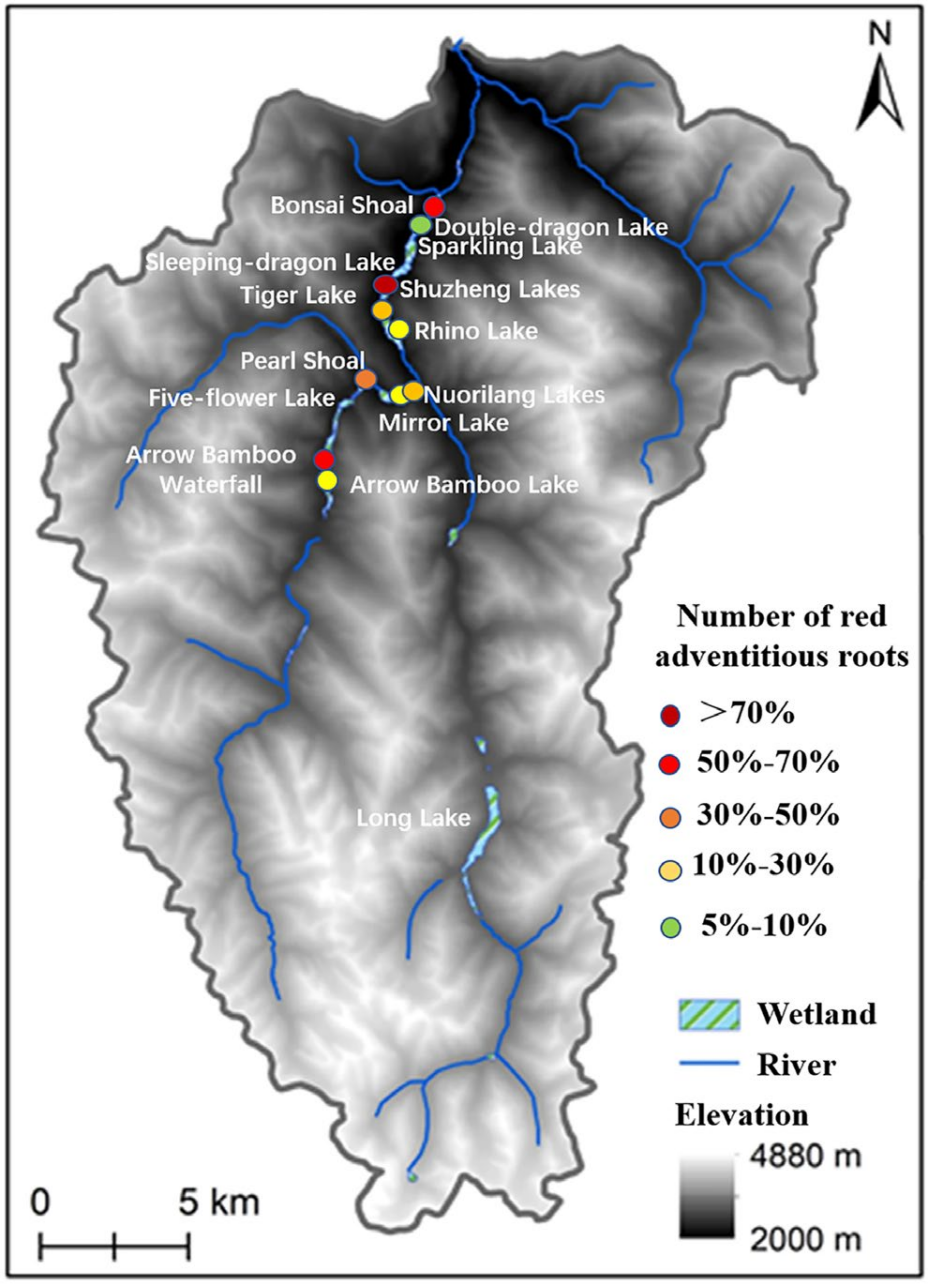
Distribution of plants with red adventitious roots in Jiuzhaigou wetland.

Occurrence of ARs exhibited distinct spatial characteristics (Figure 5 and Table S6). The plants with red ARs were primarily found in tufa wetlands, with significant differences in the number of ARs across different wetland types, where 32.2%–80.2% were in tufa shoal, 3.23%–15.2% in tufa lake, 3.20%–40.3% in tufa waterfall, and less than 10% in river areas. These plants were mainly distributed across Arrow Bamboo Lake, Pearl Shoal, Nuorilang Lakes, Shuzheng Lakes, and Bonsai Shoal. Notably, as high as 70%–90% of plants developed red ARs in the upstream and downstream areas of the Shuzheng Lakes. Among the four species with abundant ARs, *P. purdomii* was primarily distributed in the Shuzheng valley, *S. obscura* and *S. rehderiana* var. *dolia* were found largely in the areas from Shuzheng Lakes to Arrow Bamboo Lake, while *S. linearistipularis* was predominant in Bonsai Shoal.

#### 3.1.3 Morphological Characteristic

**FIGURE 6:**
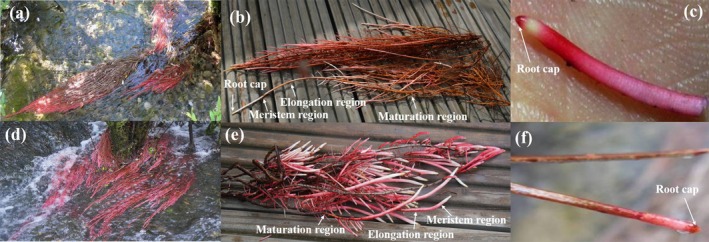
Morphological characteristics of the adventitious roots (a, d, f) and their maturation, meristem, elongation regions, and root cap characteristics (b, c, e).

ARs predominantly developed from the base of the stem, forming robust, whisker‐like roots, mostly floating on the water surface or in shallow water (Figure 6a,d). The root hairs were well developed, and this part accounted for more than half of the entire length of the floating root system (Figure 6b,e). The color of the ARs changed with the depth of the roots in water. The maturation region exposed above the water was brownish, while submerged portions were red, pink, or orange. The tender texture of the elongation region and its upward to root cap decreased and was easily broken. The meristem region was generally white or pale pink (Figure 6b,e), and the root cap was deep red (Figure 6e,f). Root samples collected in either winter or summer became reddish brown shortly after being collected from water or immediately frozen after collection (Figure S7).

Regarding the quantity and morphology of ARs (Table S6), *P. purdomii* exhibited numerous and lengthy roots, with lengths ranging from 30 to 123 cm (mean length = 50.7 cm, SD = 11.6 cm). Most *Salix* species had plentiful and relatively long, slender roots spanning 30 to 80 cm (mean length = 45.7 cm, SD = 9.7 cm). A few *Salix* plants had sparser, shorter, and finer roots, with lengths less than 30 cm (mean length = 14.6 cm, SD = 5.6 cm).

#### 3.1.4 Anatomical Characteristic

**FIGURE 7:**
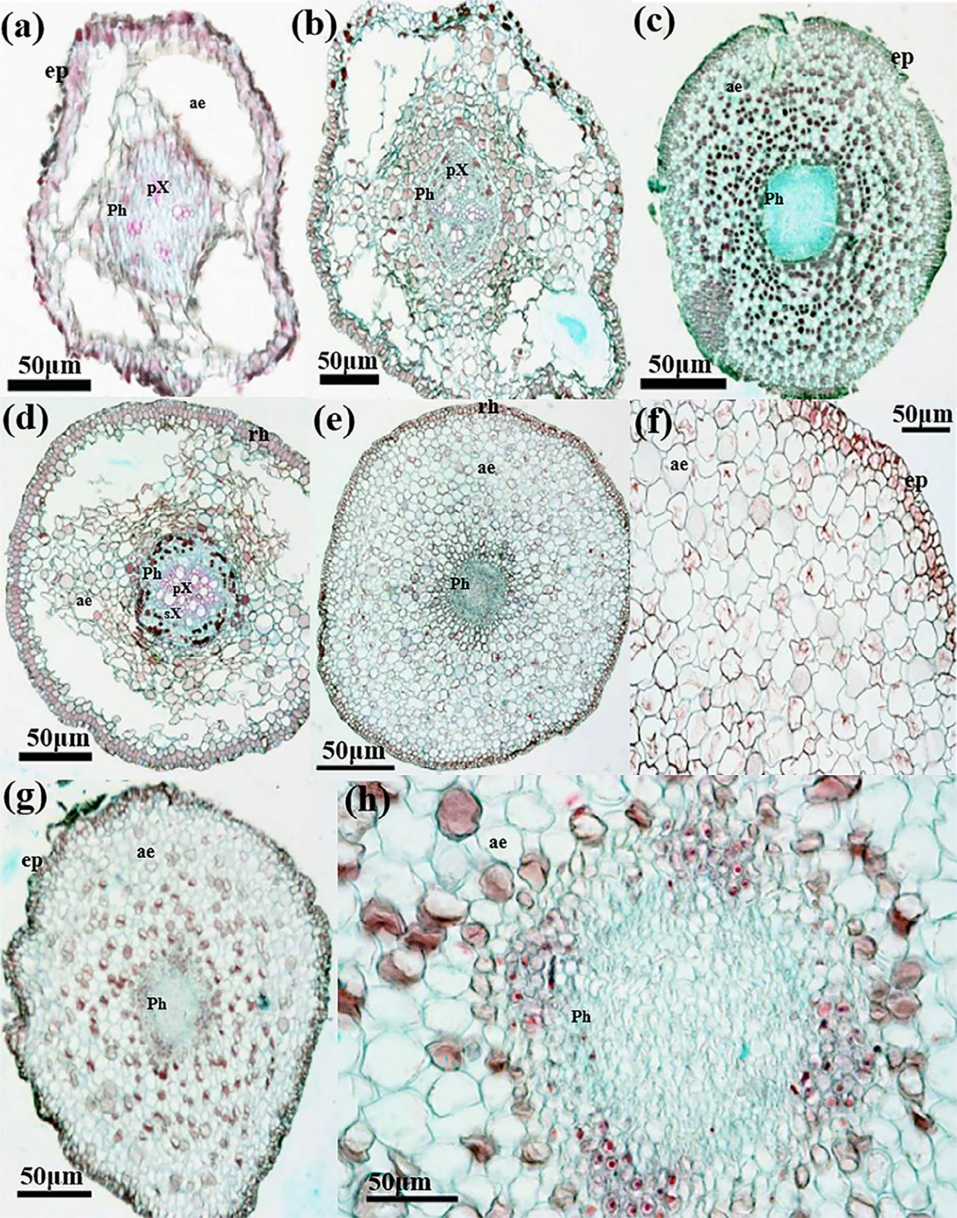
Cross section of adventitious roots of *Salix rehderiana* var. *dolia* (a–c), *Salix obscura* (d–f), and *Salix linearistipularis* (g, h). Ae, aerenchyma; ep, epidermis; Ph, phloem; pX, protoxylem; sX, secondary xylem.

The anatomical features of the three willow species were largely similar, although detailed characteristics varied among species (Table S7). Cortical cells of ARs were loosely arranged, with intercellular spaces frequently expanded to form air cavities and channels (Figure 7a,b,d). Aerenchyma, facilitating gas exchange, constituted over half of the root volume and was prominently developed. Cross‐shaped parenchyma cells were a barrier between the cortical cells and the air cavities. Apart from the exodermis, endodermis, and the 2 or 3 cell layers adjacent to the endodermis, the cortex was surrounded by cross‐shaped parenchyma cells, creating intercellular space (Figure 7c,e,g).

In the stele of ARs, xylem was either undeveloped or completely absent. The stele was undeveloped, with a thickness of aerenchyma composed of parenchyma cells in the exodermis approximately twice that of the stele.

### 3.2 Identification of Pigments

#### 3.2.1 UV–Vis and Infrared Spectra Analysis

**FIGURE 8:**
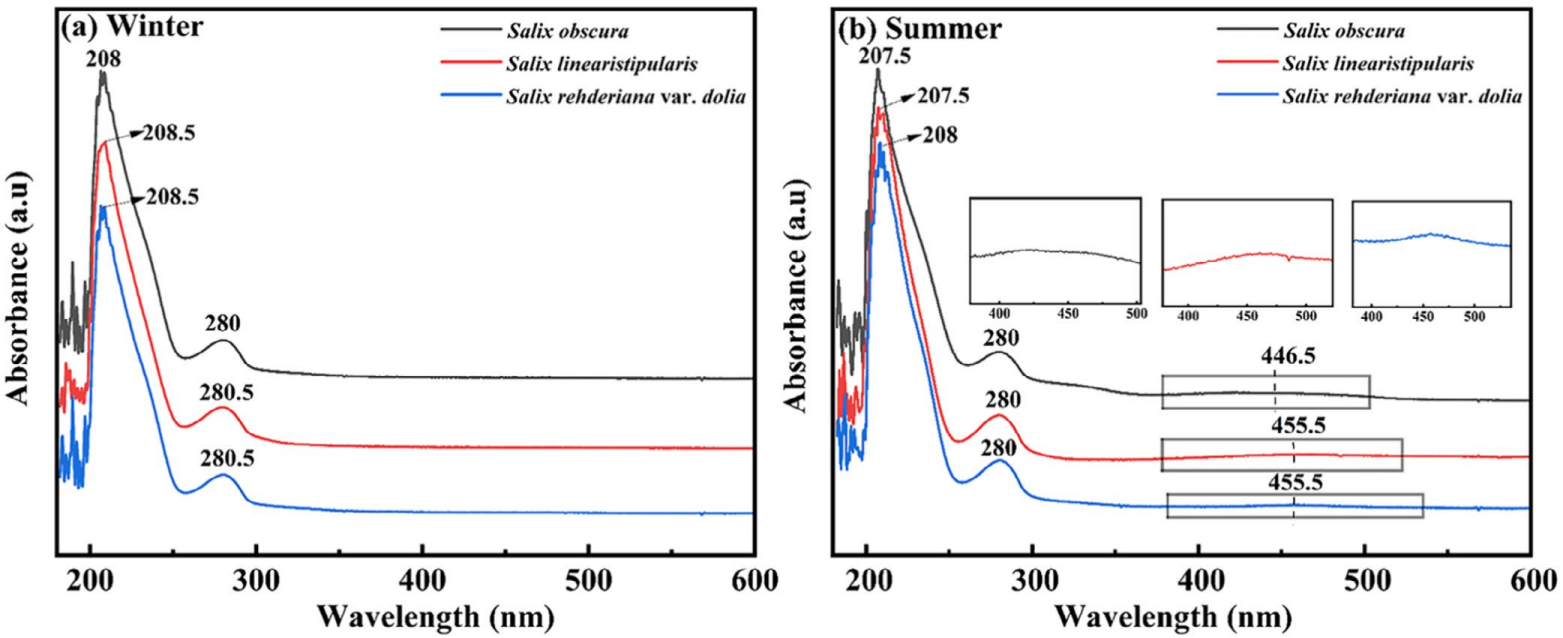
UV spectra of adventitious roots of three *Salix* species (*Salix obscura, Salix linearistipularis, Salix rehderiana* var. *dolia*) in summer and winter.

The UV–vis and infrared spectral structures of the three *Salix* species were broadly consistent, with only minor differences observed among them (Figure 8). UV–vis spectra exhibited distinct absorption peaks, with winter extracts showing two primary absorption peaks at 207.5–208 nm and 280–280.5 nm (Figure 8a), while summer extracts displayed two firm peaks at 207.0–208 nm and 280.5 nm and a weak peak at 446.5 nm or 455.5 nm (Figure 8b).

**FIGURE 9:**
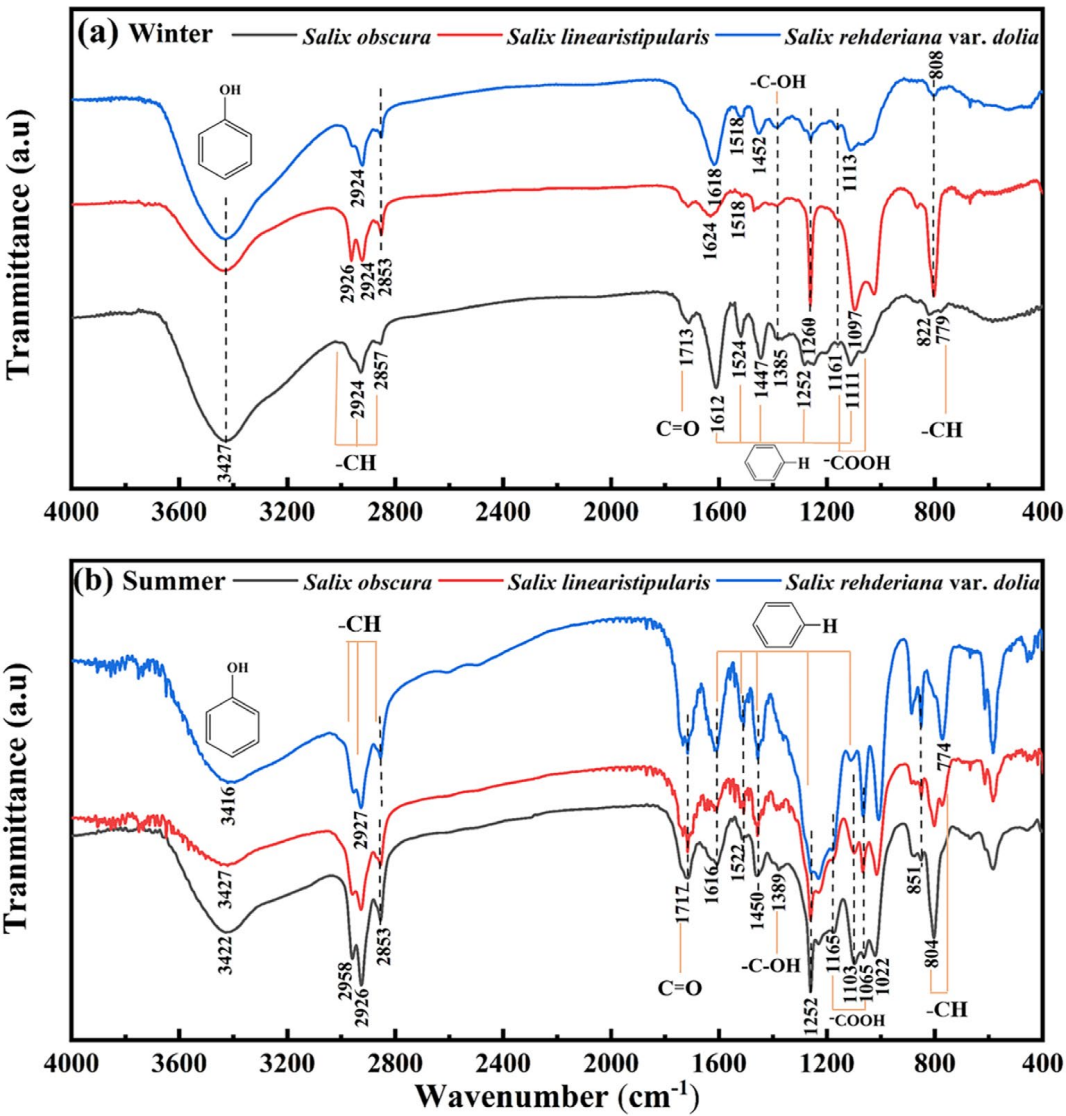
Infrared spectra of adventitious roots of three *Salix* species (*Salix obscura, Salix linearistipularis, Salix rehderiana* var. *dolia*) in summer and winter.

Infrared spectra of summer and winter roots revealed several important chemical functional groups (Figure 9a,b). Three plant species exhibited similar characteristic peaks. The strong absorption bands near 3416–3422 cm^−1^ in summer and 3427 cm^−1^ in winter samples suggested the presence of a substantial amount of phenolic hydroxyl groups (Maruf et al. 2022). The absorption peaks in 2853–2956 cm^−1^ were attributed to stretching vibrations of C−H bonds in saturated carbon structures (Guo et al. 2023). Other notable absorption bands include those at 1612–1624, 1518–1524, 1447–1452, 1252–1260, and 1097–1113 cm^−1^, which were characteristic of aromatic rings' breathing vibration modes. The absorption bands at 1161–1165 cm^−1^ and 1065 cm^−1^ were attributed to the −COOH bond (Tao et al. 2022; Wang et al. 2022). In addition, the −CO bond, −CH bond, and −COH bond were identified. The absorption bands at 804 cm^−1^, 776 cm^−1^, and 779 cm^−1^ indicated the out‐ofof‐plane bending vibrations of −CH on the benzene ring (Tao et al. 2022).

Based on analysis of the UV and IR spectra along with previous studies, the compound present in ARs was speculated potentially to be proanthocyanidins (PA) (Zhang et al. 2023; Tao et al. 2022; Saive et al. 2020). The strong absorption bands at 3427–3416 cm^−1^, 1650–1000 cm^−1^, and 850–700 cm^−1^ were likely characteristic functional groups of PA. The anthocyanin reaction of PA under hot and acidic conditions was used to test the presence of PA (Zhao et al. 2023). After adding 6% sulfuric acid to the extract and heating, both summer and winter samples turned red (Figure S8), indicating the presence of PA in the ARs.

#### 3.2.2 LC–MS Analysis

PA comprises eight monomers (Figure S9) (Alejo et al. 2020). A preliminary experiment using LC–MS revealed that *m/z* 288, *m/z* 304, and *m/z* 458 closely corresponded to the molecular weight of PA. Consequently, catechin, epicatechin, gallocatechin, and epigallocatechin gallate were selected as standards.

**FIGURE 10:**
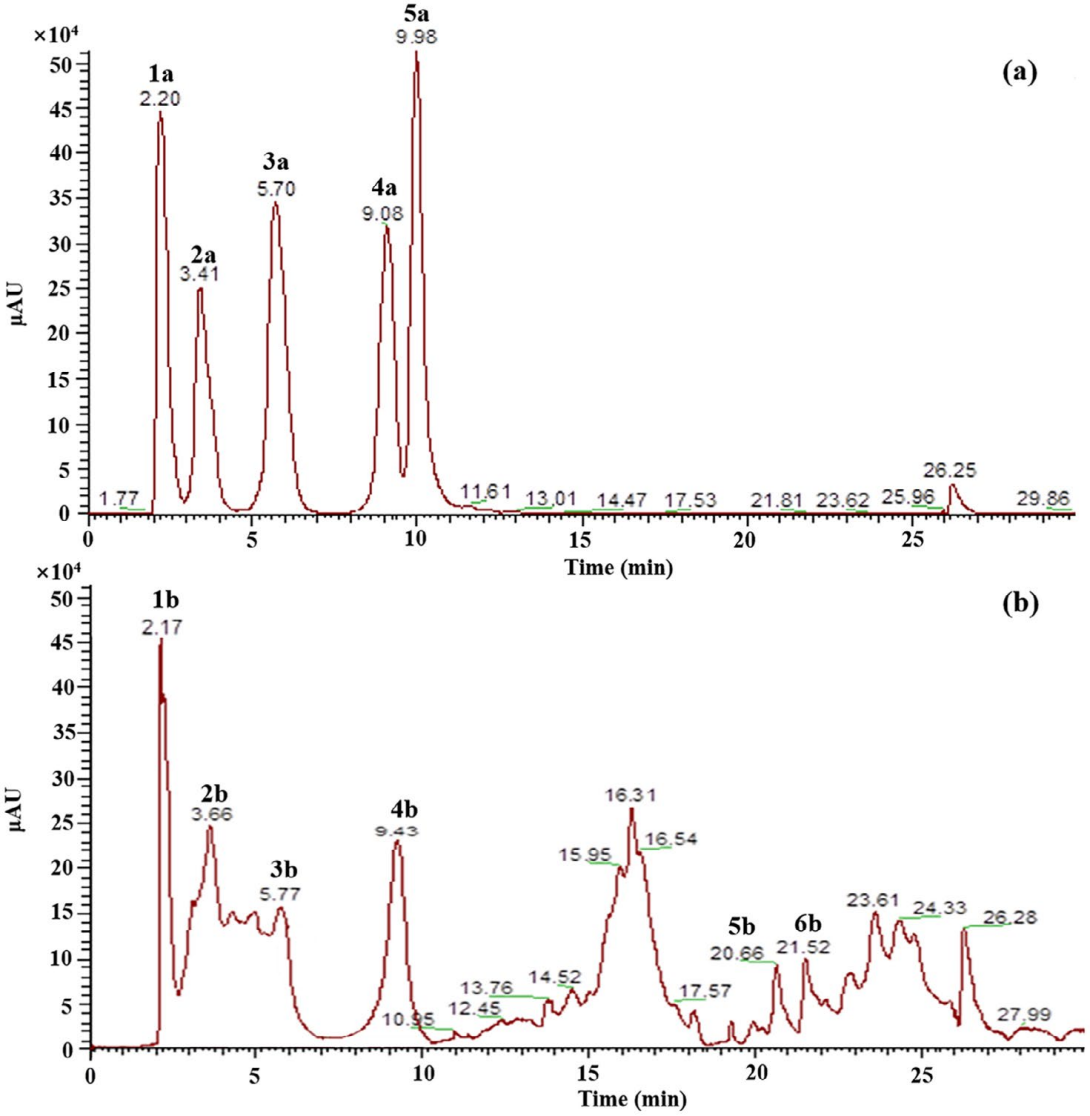
LC–MS chromatograms from methanol extract of standards (a) and adventitious roots of *Salix obscura* (b).

Table S8 illustrates the primary product ions in negative mode (*m/z*) and a tentative molecule proposed corresponding to each chromatographic peak for standards and root samples. By analyzing LC–MS data, four standards were identified as gallocatechin (3.41 min), catechin (5.70 min), epicatechin (9.08 min), and epigallocatechin gallate (9.98 min) (Figures 10a and S10a). Root samples displayed prominent ion peaks at *m/z* 304.85 (3.66 min), 288.85 (5.77 min), and 456.87 (9.43 min), corresponding to the retention time of gallocatechin, catechin, and epigallocatechin gallate. Moreover, in the mass spectra of samples (Figures 10b and S11a), the peak at *m/z* 577.12 was approximately double the value of *m/z* 289, and the peak at *m/z* 729.99 potentially corresponded to a dimer formed by *m/z* 289 and *m/z* 441. These compounds also exhibited a maximum absorbance wavelength at 280 nm (Figures S10b and S11b), a typical characteristic of PA (Fu et al. 2015; Chen et al. 2014), indicating the possible presence of two dimers of PA.

### 3.3 Color Change

#### 3.3.1 pH Effect

Color of root extract underwent significant changes in response to pH variations. The initial extract was reddish‐brown at a pH of 6.5. Upon adjustment to the pH of surface water in Jiuzhaigou (pH 8.3), the solution color intensified, and the color deepened further at a pH of 9.1. Conversely, the solution transitioned to an orange‐yellow shade in an acidic environment (pH 2.3) (Figure S12).

#### 3.3.2 Temperature and Light Irradiation Effect

**FIGURE 11:**
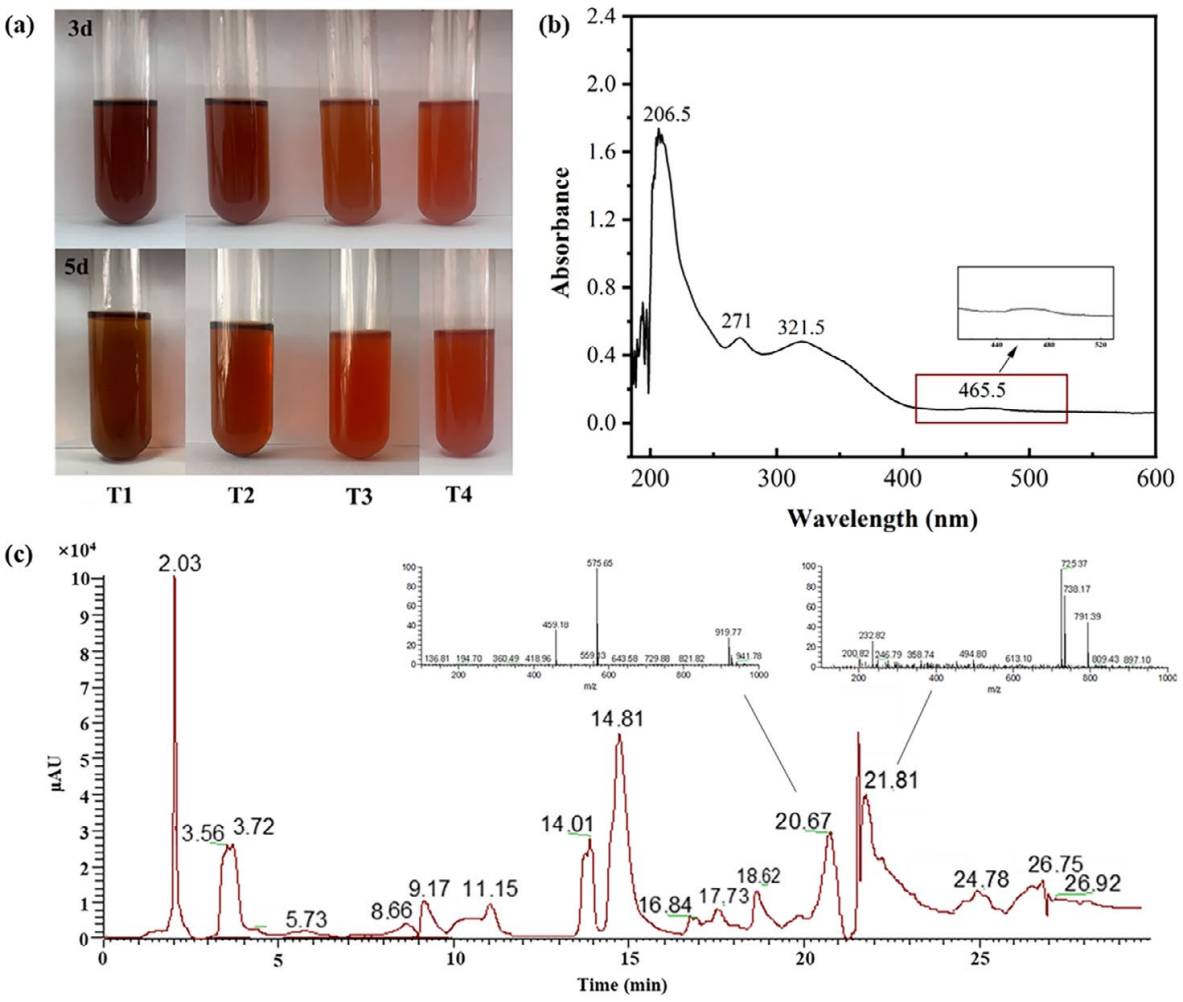
Color changes in adventitious roots of *Salix obscura* under four treatments (a), UV spectra (b), and mass spectra (c) of adventitious roots after 5 days of light irradiation.

AR extracts displayed color variations under different conditions (Figure 11a). Under room temperature light irradiation (T3), the extracts exhibited color changes by the third day and turned red by the fifth day. Higher light intensity (T4) induced red coloration by the third day. In contrast, the T1 and T2 treatments showed no significant color difference after three days. By day five, the T1 group turned brown, while the T2 group developed a light red‐brown hue.

UV spectra of the oxidized red solution revealed an enhanced absorption at 321.5 nm and a weak peak at 465.5 nm (Figure 11b). The shift in absorption wavelength was attributed to the formation of new chromophores resulting from solution oxidation. The mass spectra showed the presence of ion peaks at *m*/*z* 575.65 and *m*/*z* 725.37 (Figure 11c), which were 2 Da less than *m/z* 577.12 and 4 Da less than *m/z* 729.99.

## 4 Discussion

### 4.1 Morphological and Anatomical Characteristics of ARs

The results of this study showed that ARs in all four species emerged from the stem bases and maintained buoyancy in oxygen‐rich surface water. This adaptation was similar to that observed in other wetland plants, such as *Acorus calamus* and *Salix vinminalis*, which utilize ARs for direct oxygen acquisition and transport through aerenchyma (Mei et al. 2014; Jia et al. 2021).

Anatomical analysis revealed that *Salix* species possess well‐developed aerenchyma and degenerated mechanical tissue and xylem. The well‐developed aerenchyma is a typical characteristic of wetland plants (He et al. 2021; Mozo et al. 2021; Wang et al. 2019). This contrasts with high‐altitude *Populus* species prioritizing UV‐resistant lignification over aerenchyma development (Song and Zhang 2017), suggesting divergent evolutionary strategies within the Salicaceae driven by habitat pressures. The prominent aerenchyma development parallels adaptations in other wetland plants such as *Arundo donax, Triticum aestivum*, and *Cynodon dactylon* ; *Salix* species exhibit significantly larger air spaces (Wu et al. 2024; Yuan et al. 2022), reflecting specialization in continuous waterlogging tolerance (Bjorn et al. 2022). This anatomical predisposition explains their superior performance in wetland restoration applications, including erosion control, water purification, and sustainable biomass production (Su et al. 2016). The underdeveloped mechanical tissue indicates that ARs do not play a primary role in mechanical support but rather function in supplying oxygen and nutrients for plant respiration.

Within the study area, the species with ARs were predominantly located in the tufa wetlands of Shuzheng and Rize valleys. In addition, *Salix* plants with red ARs were observed along the Baihe rivers outside of Jiuzhaigou and Munigou tufa wetlands (unpublished data). It is plausible that there is a certain relationship between the growth of these plants and tufa. Loose, porous tufa formed by particulate matter (Qiao et al. 2022; Carthew et al. 2003) may provide structural support for plants and roots, allowing them to maintain regular physiological activities even in areas with high water flow. While tufa wetlands are rare globally, the adaptive mechanisms observed here may inform restoration strategies for degraded karst wetlands, where similar geochemical processes occur.

### 4.2 The Reason for Color Changes of ARs

Predominant pigments were proanthocyanidins, particularly notable for their ability to deepen in color under light induction (Cui et al. 2022; Hayashi et al. 2018; Gripenberg et al. 2017). In red young leaves, red willow barks, and red ARs of *Populus tremuloides* , anthocyanins were identified as primary pigments, with color changes primarily attributed to variations in pigment accumulation (Zhang et al. 2019; Zhou et al. 2022; Li et al. 2025). The difference in pigments observed in this study may be related to Jiuzhaigou's slightly alkaline water environment. Under such alkaline conditions, proanthocyanidins exhibit a red hue, while anthocyanins appear blue (Zhao et al. 2023; Xue et al. 2024). This highlights genus‐specific pigment adaptation to local environmental constraints.

Upon light exposure, the UV spectra of the irradiated extract displayed an absorption peak at 321.5 nm, indicating the formation of carbonyl groups conjugated with benzene rings. Weak peaks in the 400–500 nm range suggest the oxidation of phenolic hydroxyl groups in the root extract to quinone structures. Mass spectra revealed peaks at *m*/*z* 575.65 and *m*/*z* 725.37, corresponding to a loss of 2 Da and 4 Da from *m*/*z* 577.12 and *m*/*z* 729.99, respectively. This indicates the formation of intramolecular and aldehyde group bonds (−C=O), suggesting the products of dehydrogenation and oxidation of dimeric polyphenolic compounds. This mechanism is similar to the color deepening observed during the storage of red rice (Hayashi et al. 2018). Comparative analysis of the UV spectra of ARs revealed a weak peak at 444.5 nm in summer samples, indicating the presence of quinone compounds, which supports our hypothesis that color changes in ARs result from internal compound transformations. In the visible light range of 420 nm to 580 nm, absorbed colors shift from blue to green, while reflected colors (complementary colors) range from yellow to red (Qiu and Guo 2021; Xu et al. 2024), which is the main reason for the increase in the a* and b* values and the deepening of color during the warm season. However, due to the limited sample size of ARs, proanthocyanidins quantification was not performed in this study. Whether pigment accumulation also changes during the color transformation process will be addressed in our future research.

After harvest and removal from their aquatic habitat, the ARs underwent a notable color shift from red to brown. This change is similar to the post‐harvest browning observed in litchi, which is attributed to the enzymatic oxidation of catechins and other polyphenolic constituents, forming brown pigments (Liu et al. 2010). To mitigate such discoloration in our study, we promptly submerged the harvested roots in containers filled with surface water from Jiuzhaigou. This method preserved the roots, maintaining sample integrity until analysis and avoiding unwarranted biochemical changes that might arise from suboptimal storage conditions.

### 4.3 Seasonal Influences on ARs Pigmentation and Their Bioindicator Potential

Our findings revealed that chromatic variations in ARs are phenologically synchronized with seasonal environmental parameters. Multivariate regression analysis demonstrated significant correlations with air temperature, precipitation, and NO_3_^−^ concentration. However, no chromatic changes were observed when NaNO_3_ solutions were added in gradients, suggesting indirect mediation through coupled environmental variables rather than direct ionic regulation. Notably, light and temperature emerged as key drivers of pigment transformation. Light intensity and spectral composition are known to regulate both primary and secondary metabolite biosynthesis, particularly influencing flavonoid production pathways (Thoma et al. 2020; Qin et al. 2024), while temperature modulates oxidation rates of PA (Lin et al. 2021). This aligns with our observations that elevated temperatures and irradiance accelerated pigment oxidation, inducing seasonal reddening (Figure 11a). Conversely, light‐deprived ARs exhibited whitening (Li et al. 2025), further underscoring light as a critical factor.

The dynamic chromic response of ARs highlights their potential as bioindicators for real‐time environmental monitoring. Their advantages include high temporal resolution, cost efficiency, and scalability. These attributes make them particularly suited for large‐scale assessments of climate change impacts in heterogeneous wetland ecosystems, where traditional monitoring often struggles with temporal resolution and spatial coverage. For instance, ARs could serve as proxies for tracking microclimatic changes in tufa wetlands, where traditional sensors face logistical challenges. In addition, their color shifts may reflect cumulative thermal stress or UV exposure, aiding in early warnings of ecosystem degradation under climate change.

However, practical implementation requires addressing inherent limitations. First, the current findings are constrained by small sample sizes and species specificity, limiting extrapolation to other wetland systems. Second, ARs' high metabolic activity may inadvertently alter local nutrient cycling, posing ecological risks if deploy at scale. Third, color responses could be confounded by co‐varying factors, such as water chemistry and microbial interactions. Future research should prioritize multi‐scale validation, expanding sampling to diverse species and biogeographic regions to access universal applicability. Integrating metabolomic profiling with environmental data would elucidate mechanistic links between pigment and abiotic stressors. Furthermore, controlled mesocosm experiments could disentangle the synergistic effects of temperature, light, and nutrient fluxes on chromatic responses. Such advancements would enhance ARs' utility as a bioindicator, providing actionable insights for conserving Jiuzhaigou's tufa wetlands and analogous karst ecosystems globally.

## 5 Conclusion

This study systematically analyzed the adaptive characteristics and color alteration mechanisms of adventitious roots in the tufa wetlands of Jiuzhaigou. The results indicate that these roots have evolved morphological and anatomical strategies to cope with waterlogged environments, enhancing oxygen and nutrient acquisition under hypoxic conditions. The color changes in adventitious roots, which are responsive to temperature and light intensity variations, suggest their potential as bioindicators for environmental monitoring. They could be particularly useful in forecasting and assessing the impacts of climate change on wetland ecosystems. Future studies could quantify metabolic products within these roots and explore additional environmental factors to fully understand the pigment transformation mechanisms and their role in response to environmental changes.

## Author Contributions

**Ting Liu:** data curation (lead), investigation (lead), methodology (lead), writing – original draft (lead). **Junhuai Xu:** data curation (equal), methodology (equal). **Weiyang Xiao:** data curation (equal), investigation (equal). **Lv Zhou:** data curation (equal), investigation (equal). **Sha Deng:** methodology (equal). **Yingzhou Chen:** investigation (equal). **Xue Qiao:** funding acquisition (equal), writing – review and editing (equal). **Zongliang Du:** methodology (equal), writing – review and editing (equal). **Ya Tang:** conceptualization (lead), funding acquisition (lead), writing – review and editing (lead).

## Conflicts of Interest

The authors declare no conflicts of interest.

## Supporting information


**Data S1.** Supporting Information.

## Data Availability

The data that supports the findings of this study are available in the Supporting Information.
