# Supplementary material for: Seasonal Coloration and Ecological Adaptations of Adventitious Roots of Four Salicaceous Species in Jiuzhaigou World Natural Heritage Site, Southwestern China
Source: Ecol Evol. 2025 Apr 15;15(4):e71218. doi: 10.1002/ece3.71218 (PMC12000238; doi:10.1002/ece3.71218)
Supplement: Supplementary file 1 — Data S1. Supporting Information. [file ECE3-15-e71218-s001.pdf]

Supporting information

Seasonal coloration and ecological adaptations of adventitious roots of four Salicaceous species in Jiuzhaigou World Natural Heritage Site, southwestern China

Ting Liu[1, 2], Junhuai Xu[3, 4], Weiyang Xiao[5], Lv Zhou[5], Yingzhou Chen[2], Xue Qiao[1], Sha Deng[4], Zongliang Du[4], Ya Tang[2, *]

[1]Institute of New Energy and Low-carbon Technology, Sichuan University, No. 24, South Section One, First Ring Road, Chengdu 610065, Sichuan, China

[2]College of Architecture and Environment, Sichuan University, No. 24, South Section One, First Ring Road, Chengdu 610065, Sichuan, China

[3]China Southwest Architecture Design and Research Institute Co. Ltd., CSCEC Green Construction Engineering Research Center, Chengdu 610041, Sichuan, China

[4]College of Biomass Science and Engineering, Sichuan University, No. 24, South Section One, First Ring Road, Chengdu 610065, Sichuan, China

[5]Jiuzhaigou Administrative Bureau, Zhangzha Town, Jiuzhaigou County 623402, Sichuan, China

*Ya Tang, corresponding author, tangya@scu.edu.cn

Table S1. Monitoring sites for seasonal color variation of adventitious roots in four Salicaceous species

Number	Species	Sampling site	Latitude (°N)	Longitude (°E)	Elevation (m)	Monitoring frequency
1	*Salix rehderiana* var. *dolia*	Arrow Bamboo Lake	33.1386	103.8731	2660	Once a month
2	*Salix obscura*	Shuzheng Lakes	33.1990	103.8948	2267	
3	*Salix linearistipularis*	Bonsai Shoal	33.2234	103.9107	2180	
4	*Populus purdomii*	Shuzheng Lakes	33.1989	103.8950	2273	

Table S2. Locations of water chemistry monitoring in Jiuzhaigou

Number	Monitoring point	Wetland type	Latitude (°N)	Longitude (°E)	Elevation（m）
1	Arrow Bamboo Lake	Tufa lake	33.1386	103.8731	2660
2	Arrow Bamboo Waterfall-1	Tufa waterfall	33.1408	103.8704	2658
3	Arrow Bamboo Waterfall-2	Tufa waterfall	33.1376	103.8725	2656
4	Shuzheng Lakes-1	Tufa shoal	33.1989	103.8950	2273
5	Shuzheng Lakes-2	Tufa shoal	33.1990	103.8948	2267
6	Shuzheng Lakes-3	Tufa shoal	33.1992	103.8942	2252
7	Bonsai Shoal-1	Tufa shoal	33.2235	103.9103	2180
8	Bonsai Shoal-2	Tufa shoal	33.2234	103.9107	2182
9	Bonsai Shoal-3	Tufa shoal	33.2231	103.9105	2181

Table S3. Water chemistry parameters and measurement methods

**Parameters**	**Unit**	**Monitoring frequency**	**Methods**	**Analysis site**	**Instrument**
pH			Glass electrode method	Field	WTW Multi 3620
Conductivity	μS/cm		Electrode method	Field	WTW Multi 3620
Temperature	℃		Electrode method	Field	WTW Multi 3620
DO	mg/L	Once a month	Electrochemical probe method	Field	WTW Multi 3620
Water velocity	m/s		Electrode method	Field	Xin
Ion concentration	mg/L		Ion Chromatography	Laboratory	LS 1206B Current Meter

Table S4. Sampling information for anatomical analysis and pigment extraction of *Salix* species

Number	Species	Sampling site	Latitude (°N)	Longitude (°E)	Elevation (m)	Sampling type	Collect time
1	*Salix rehderiana* var. *dolia*	Arrow Bamboo Lake	33.1386	103.8731	2660	RTs; ARs	RTs：2022.07.09<br>ARs:<br>Winter: 2021.12.22<br>Summer2022.08.13
2	*Salix rehderiana* var. *dolia*	Arrow Bamboo Waterfall	33.1408	103.8704	2658	RTs; ARs	
3	*Salix rehderiana* var. *dolia*	Arrow Bamboo Waterfall	33.1486	103.8715	2657	RTs; ARs	
4	*Salix obscura*	Shuzheng Lakes	33.1992	103.8942	2252	RTs; ARs	
5	*Salix obscura*	Tiger Lake	33.1913	103.8915	2323	RTs; ARs	
6	*Salix linearistipularis*	Bonsai Shoal	33.2234	103.9107	2182	RTs; ARs	

RTs: root tips; ARs: adventitious roots

Table S5. Monthly variations of L^*, a^*, b^*, and C^* in the maturation region, elongation region, and meristem region of adventitious roots of *Salix obscura* from March 2021 to February 2022.

Time	Root region	L^*	a^*	b^*	C^*	Color
2021.03	MA	34	7	7	9.90	
	EL	38	12	7	13.9	
	ME	32	15	5	15.8	
2021.04	MA (Emergent)	87	0	2	2.00	
	MA (Submerged)	49	12	8	14.4	
	EL	43	18	12	21.6	
	ME	47	16	11	19.4	
2021.05	MA	50	15	12	19.2	
	EL	48	16	16	22.6	
	ME	41	17	21	27.0	
	New root	54	47	24	52.8	
2021.07	MA	39	17	9	19.2	
	EL	48	14	16	21.3	
	ME	59	43	45	62.2	
	New root	47	60	25	65.0	
2021.08	MA	45	38	32	49.7	
	EL	44	38	29	47.8	
	ME	41	43	34	54.8	
2021.09	MA	48	62	51	80.3	
	EL	50	64	52	82.5	
	ME	61	57	35	66.9	
2021.10	MA	47	48	49	68.6	
	EL	50	50	49	70.0	
	ME	51	56	49	74.4	
2021.11	MA	49	26	29	38.9	
	EL	47	16	17	23.3	
	ME	49	13	10	16.4	
2021.12	MA	41	17	8	18.8	
	EL	39	23	11	25.5	
	ME	48	23	19	29.8	
2022.02	MA (Emergent)	69	2	5	5.39	
	MA (Submerged)	41	10	10	14.1	
	EL	39	15	7	16.6	
	ME	40	11	9	14.2	

MA: Maturation region of adventitious roots
EL: Elongation region of adventitious roots
ME: Meristem region of adventitious roots

Table S6. Proportions of plants with adventitious roots and their lengths for four Salicaceous species in Jiuzhaigou wetlands

Monitoring point	Wetland type	Ratio of plants with ARs (%)	Ratio of species of ARs plants (%)				ARs length (cm)			
			S. rehderiana* var. *dolia	***S. obscura***	***S. linearistipularis***	***P. purdomii***	***S. rehderiana* var. *dolia***	***S. obscura***	***S. linearistipularis***	***P. purdomii***
Arrow Bamboo Lake	Tufa shoal	40.3	65.5	30.2	0	3.3	31.3±5.1	35.2±6.7	/	31.6±4.4
Arrow Bamboo Waterfall	Tufa waterfall	37.6	79.2	15.7	0	5.1	35.8±9.4	23.6±5.1	/	60.2±8.8
Pearl Shoal	Tufa shoal	26.2	61.4	38.6	0	0	15.2±4.1	13.9±3.6	/	/
Pearl Shoal-Mirror Lake River	Tufa river	2.51	100	0	0	0	8.78±2.22	/	/	/
Mirror Lake	Tufa lake	9.81	53.2	46.8	0	0	21.3±2.5	31.2±17.6	/	/
Nuorilang Lakes	Tufa shoal	32.2	61.3	38.7	0	0	30.3±6.4	43.2±8.8	/	/
Nuorilang waterfall	Tufa waterfall	3.20	100	0	0	0	28.9±9.7	/	/	/
Rhino Lake	Tufa lake	15.2	36.0	61.2	0	2.80	35.6±13.3	44.7±6.3	/	43.6±5.4
Tiger Lake shoal	Tufa shoal	36.7	28.2	68.6	0	3.2	38.1±11.4	35.6±5.5	/	47.2±9.8
Shuzheng waterfall	Tufa waterfall	20.3	12.4	51.4	0	36.2	23.2±8.5	32.7±8.3	/	43.8±11.2
Shuzheng Lakes	Tufa shoal	80.2	16.2	77.3	0	6.5	41.2±13.7	53.4±11.5	/	61.7±13.3
Sleeping-dragon Lake	Tufa lake	3.23	0	100	0	0	/	13.4±12.7	/	/
Sparkling Lake	Tufa lake	5.32	12.6	87.4	0	0	14.5±2.5	24.4±6.3	/	/
Double-dragon Lake	Tufa lake	6.73	0	65.0	35.0	0	/	37.2±11.5	32.3±5.6	/
Bonsai Shoal	Tufa shoal	62.5	0	13.4	83.4	3.2	/	58.2±13.8	46.5±7.4	66.8±12.2

ARs: adventitious roots

Table S7. Anatomical characteristics of adventitious roots in *Salix* species

**Index**	***Salix rehderiana* var. *dolia***	***Salix obscura***	***Salix linearistipularis***
Xylem thickness (μm)	2.12	3.46	0
Phloem thickness (μm)	3.83	2.95	2.34
Aerenchyma thickness (μm)	39.4	41.2	40.2
Ratio of xylem (%)	3.31	5.25	0.11
Ratio of phloem (%)	13.2	11.4	12.2
Ratio of aerenchyma (%)	61.7	54.5	53.9

Table S8. The pigments components of standards and adventitious roots of *Salix obscura*.

Peak		Retention Time （min）	λ_{max}/ nm	$[M-H]^-$ (*m/z*)	Molecular formula	Identification
Standard	1	2.20	249	174.84	$C_6H_8O_6$	L-Ascorbic acid
	2	3.41	270	304.85	$C_{15}H_{14}O_7$	(-)-Gallocatechin
	3	5.70	276	288.85	$C_{15}H_{14}O_6$	(-)-Catechin
	4	9.08	277	288.85	$C_{15}H_{14}O_6$	(-)-Epicatechin
	5	9.98	274	456.88	$C_{22}H_{18}O_{11}$	(-)-Epigallocatechin gallate
Adventitious roots	1	2.17	249	174.84	$C_6H_8O_6$	L-Ascorbic acid
	2	3.66	274	304.84	$C_{15}H_{14}O_7$	Gallocatechin
	3	5.77	279	288.85	$C_{15}H_{14}O_6$	Catechin
	4	9.43	274	456.87	$C_{22}H_{18}O_{11}$	Epigallocatechin gallate
	5	17.57	274	729.22	$C_{27}H_{30}O_{16}$	Diploid
	6	21.52	274	577.12	$C_{30}H_{26}O_{12}$	Diploid

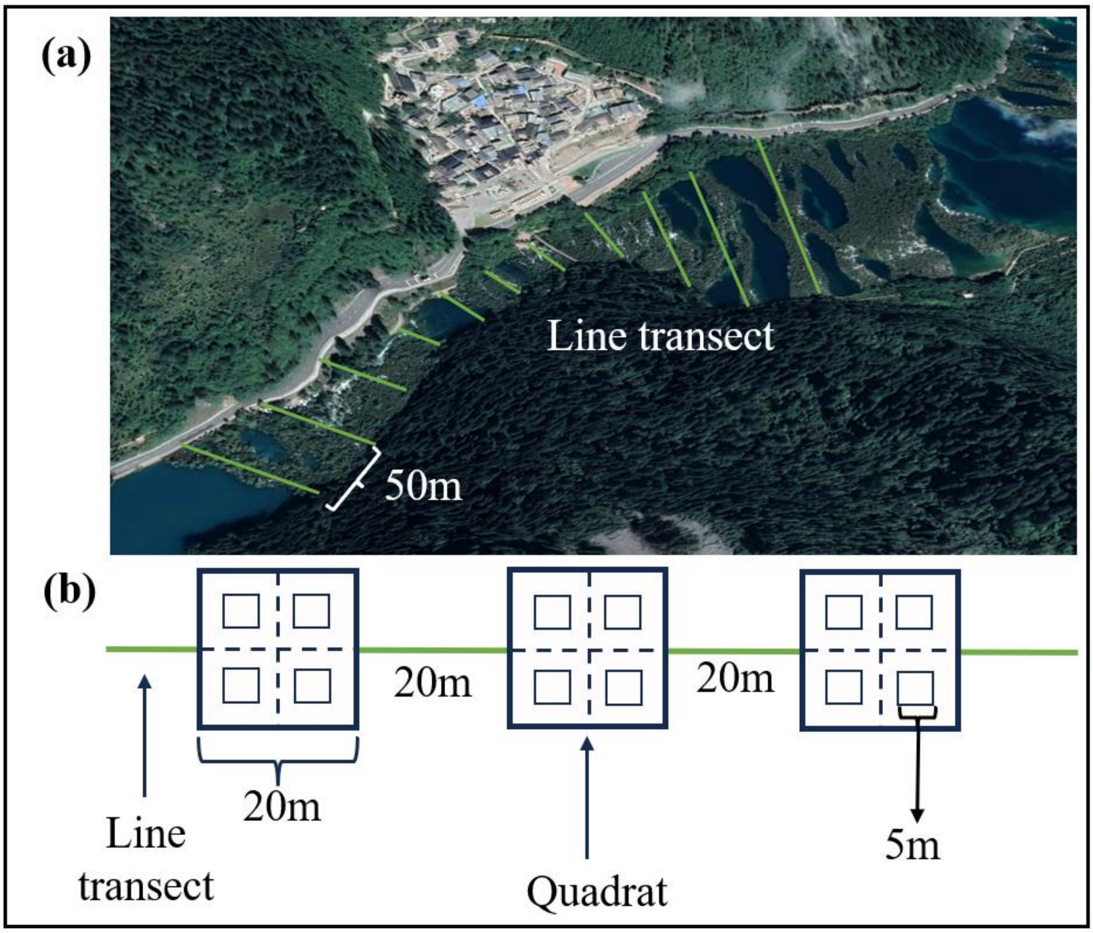

Figure S1. Diagram of quadrats for the survey of plant spatial distribution in Jiuzhaigou wetlands.

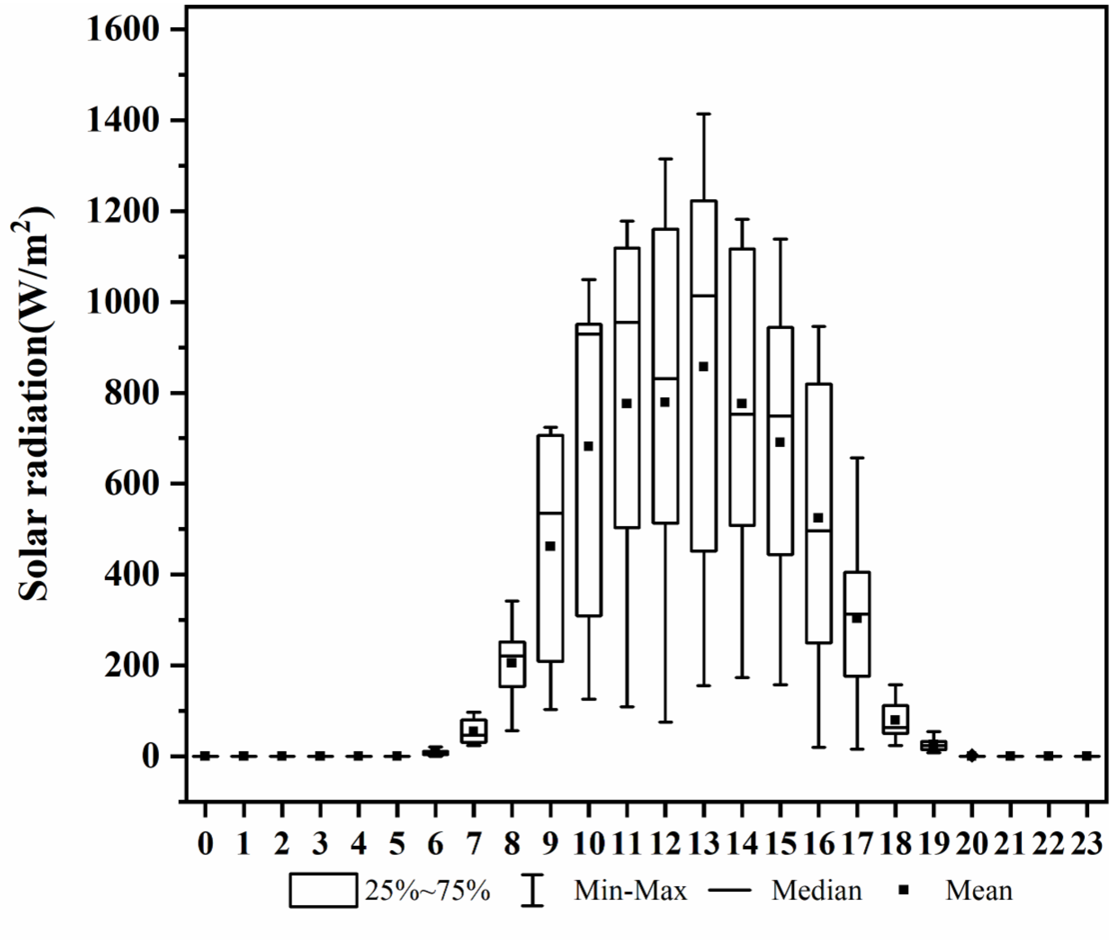

Figure S2. Daily variation of solar radiation intensity at Zezhawa in July 2021.

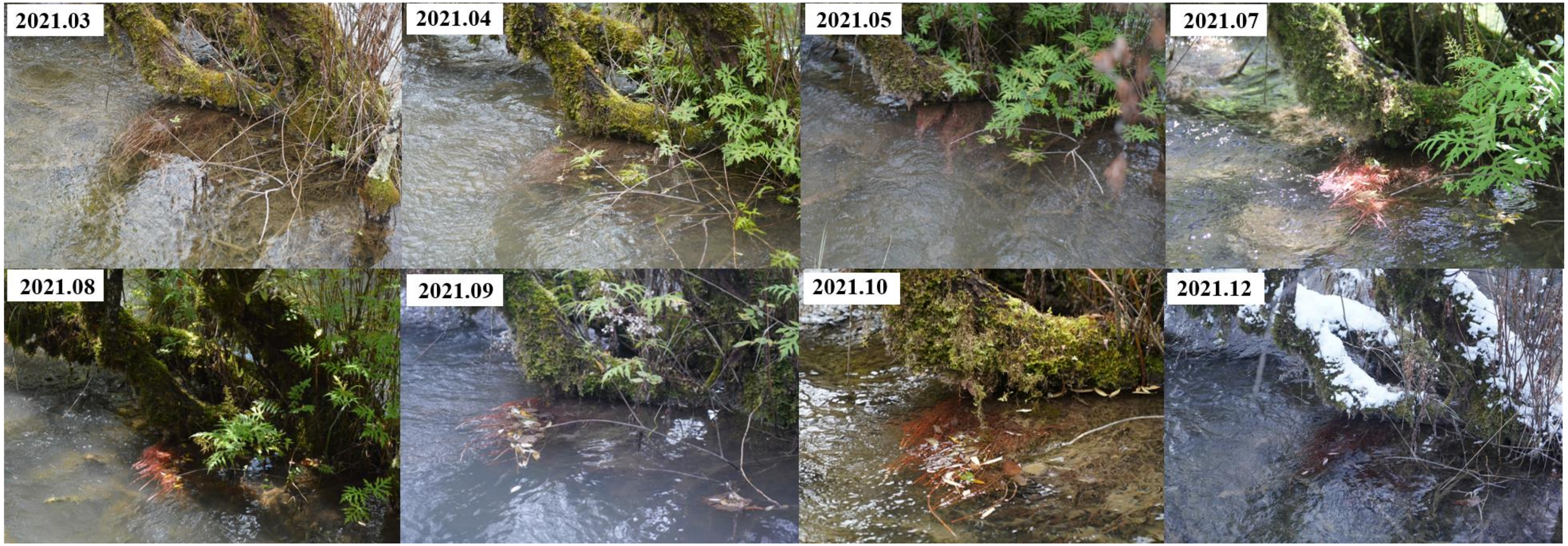

Figure S3. Color changes in adventitious roots of *Salix rehderiana* var. *dolia*.

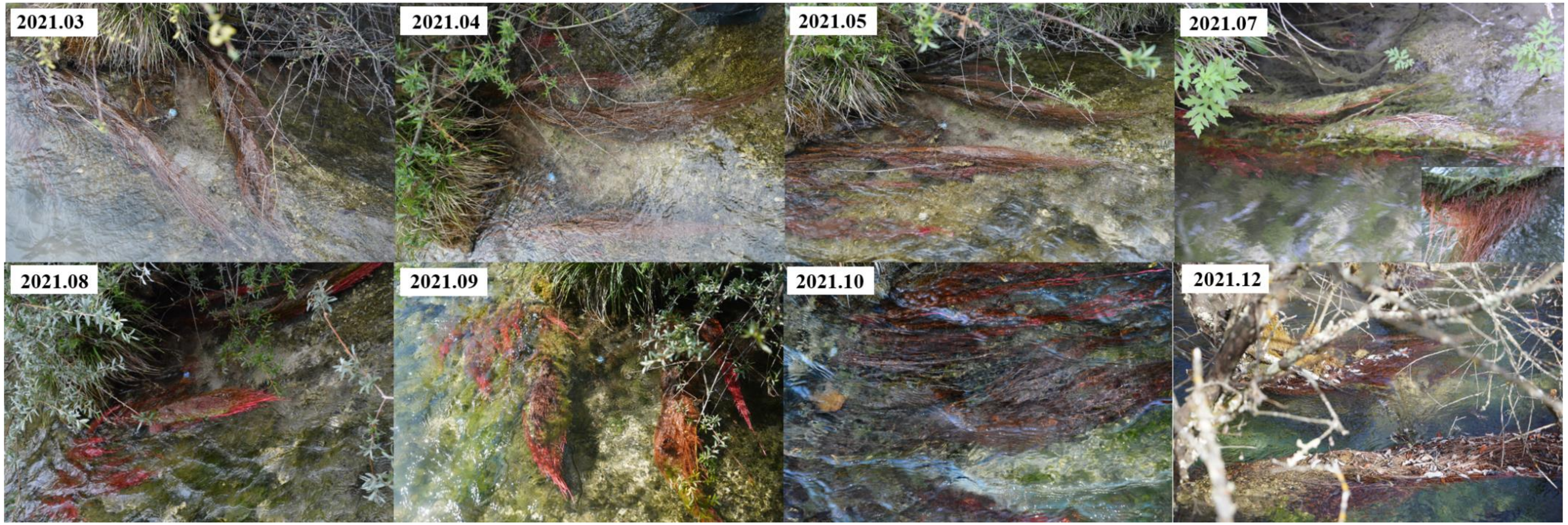

Figure S4. Color changes in adventitious roots of *Salix linearistipularis*.

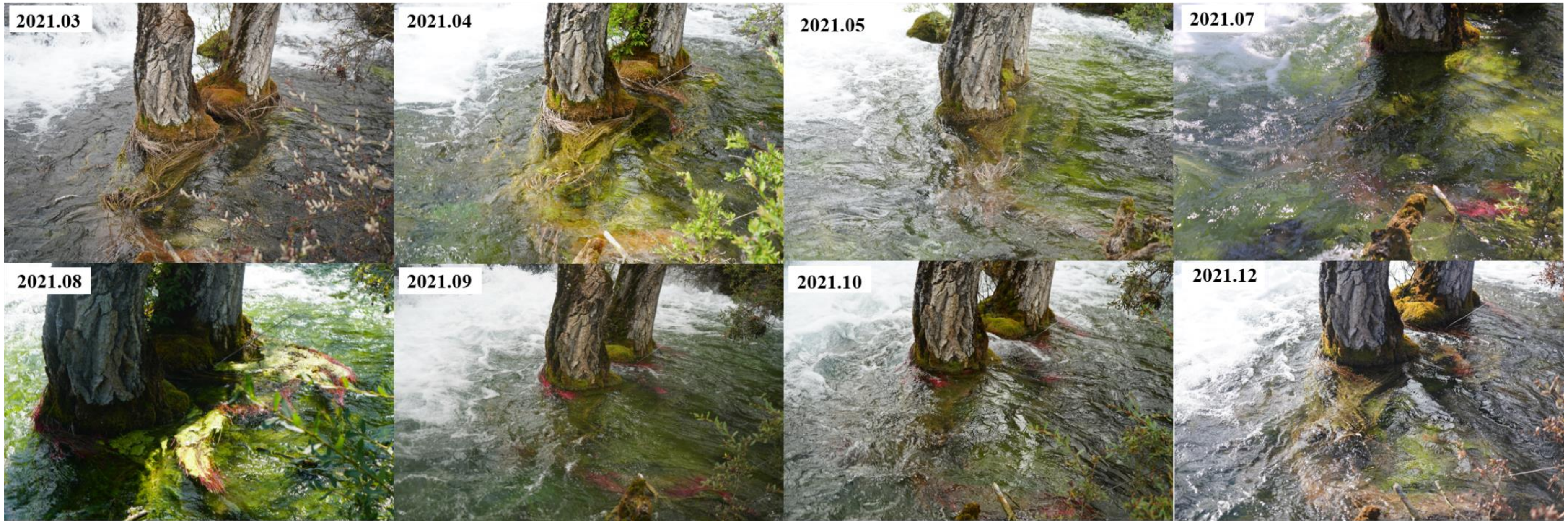

Figure S5. Color changes in adventitious roots of *Populus purdomii*.

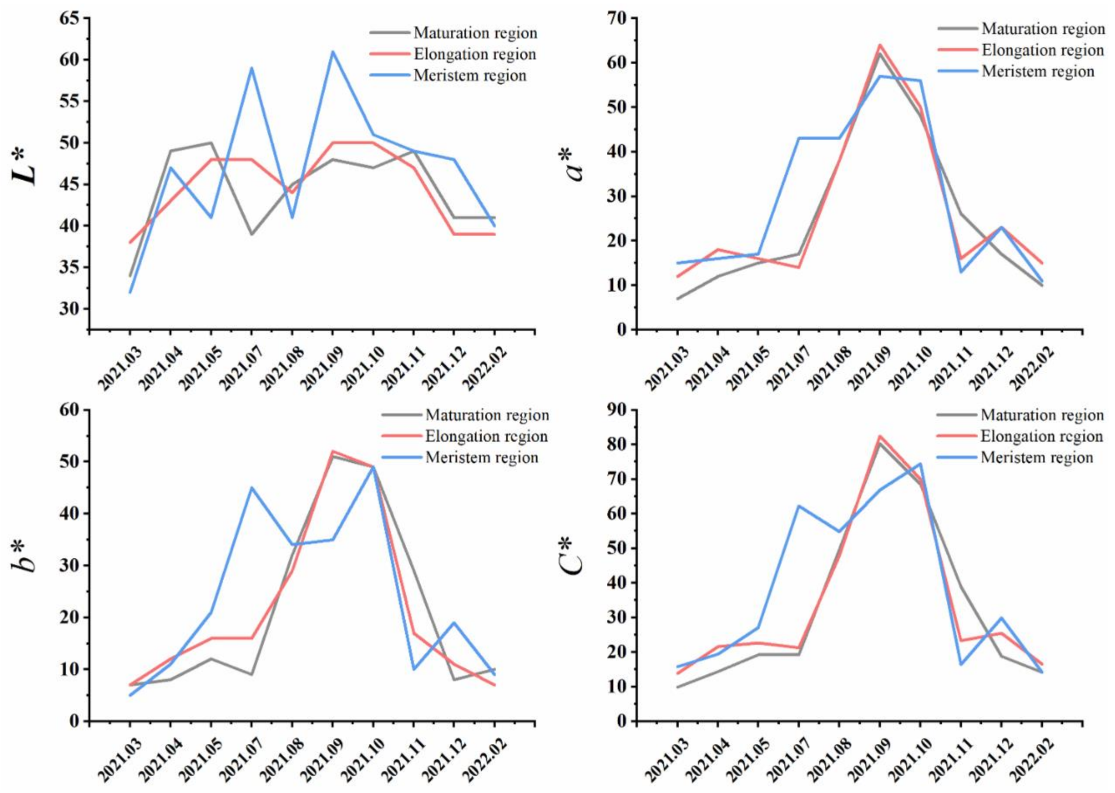

Figure S6. Variations in the luminosity (*L**), red/green (*a**), yellow/blue (*b**), and Chromaticity (*C**) of adventitious roots of *Salix obscura* from March 2021 to February 2022.

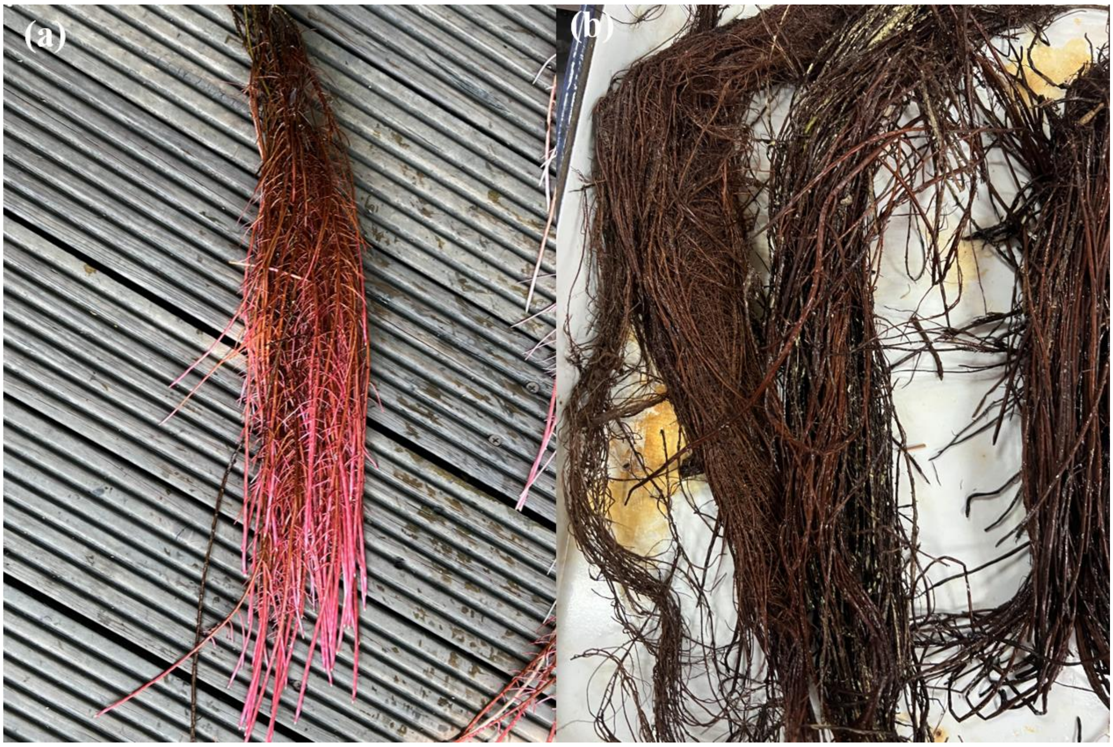

Figure S7. The color of adventitious roots after harvesting (a) and freezing (b).

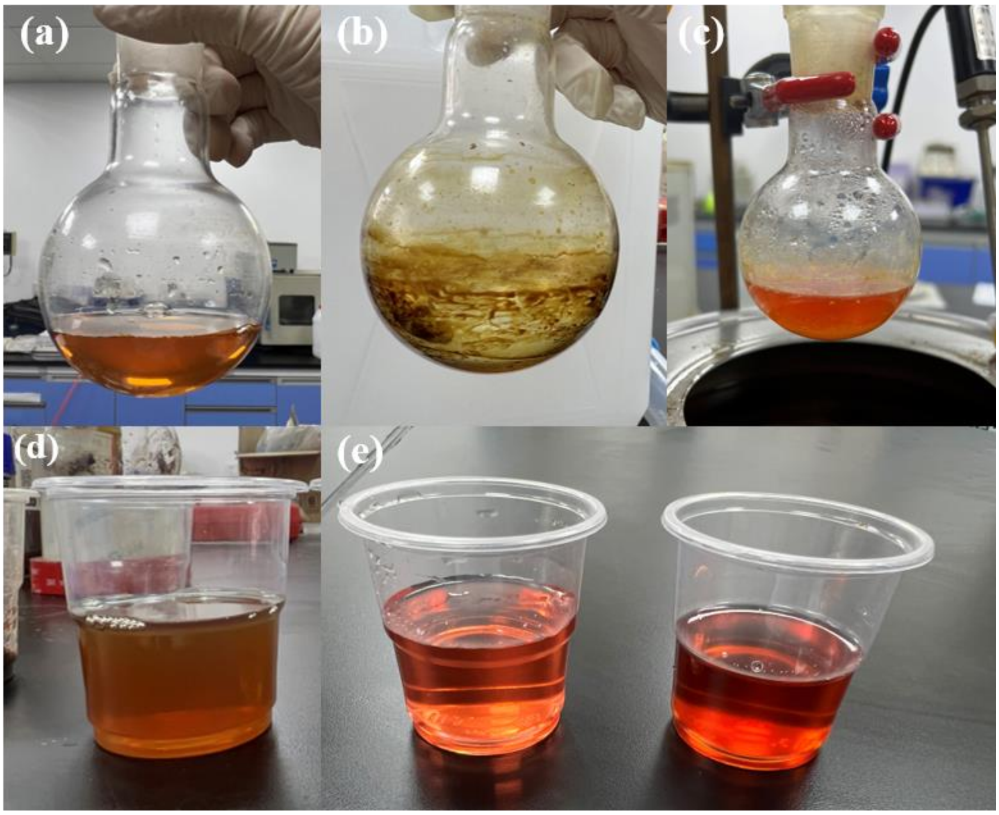

Figure S8. Adventitious root extracts (a, d), concentrate after rotary evaporation (b), and solution after treatment with 6% sulfuric acid (c, e).

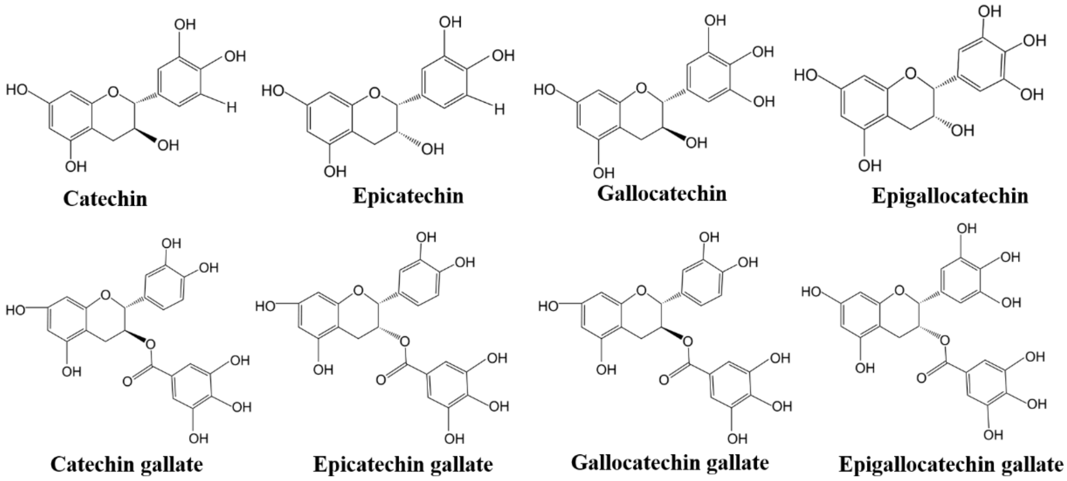

Figure S9. Chemical structures of proanthocyanidin monomers.

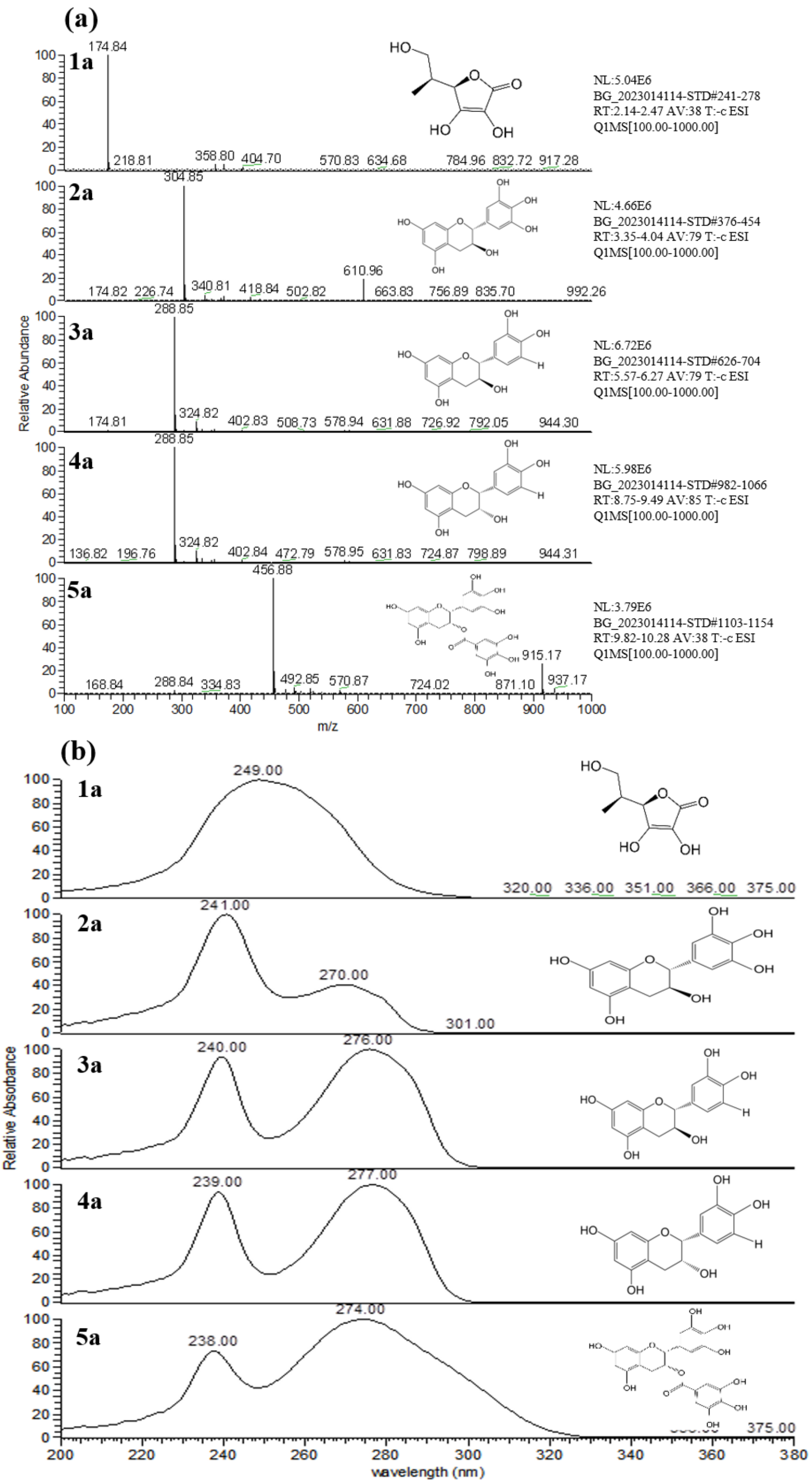

Figure S10. Mass spectra of standards (a) and their UV spectra of the peaks (b).

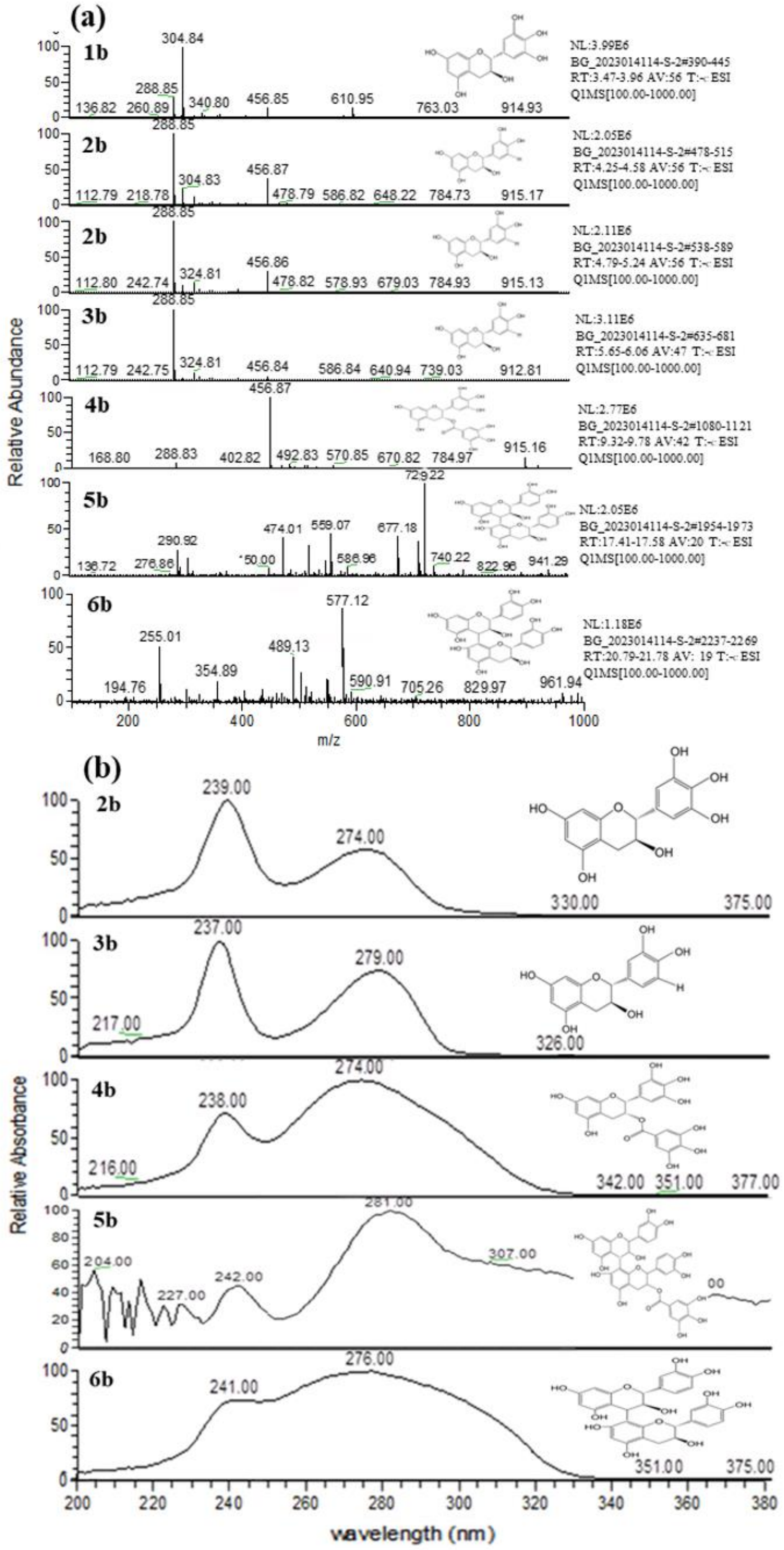

Figure S11. Mass spectra of adventitious root samples (a) and their UV spectra of the peaks (b).

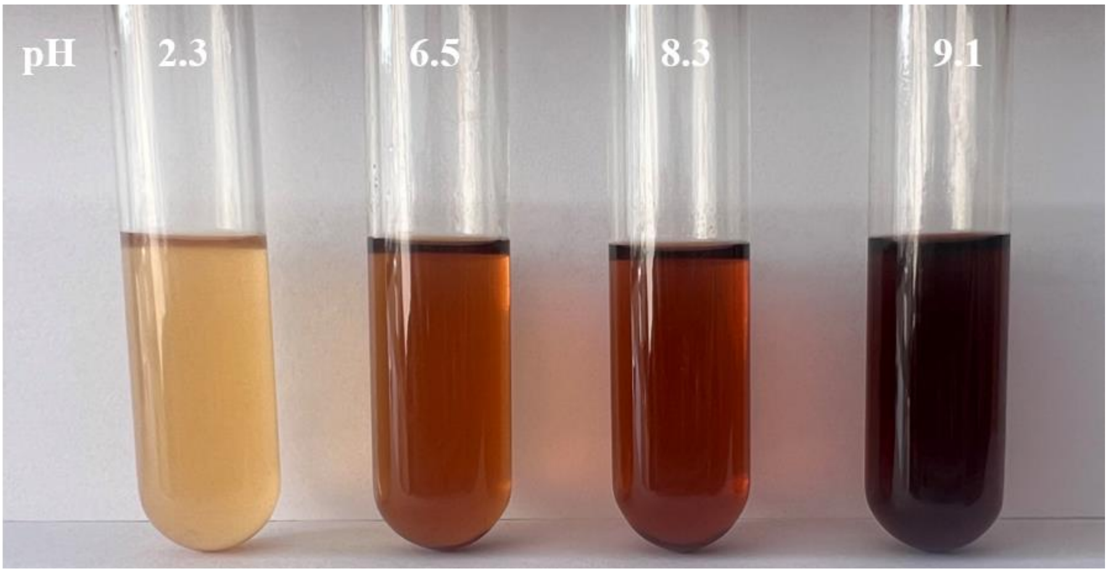

Figure S12. The color of adventitious root extracts at different pH value.
